# Systematic Approach Revealed *SERPING1* Splicing-Affecting Variants to be Highly Represented in the Czech National HAE Cohort

**DOI:** 10.1007/s10875-023-01565-w

**Published:** 2023-08-25

**Authors:** Hana Grombirikova, Viktor Bily, Premysl Soucek, Michal Kramarek, Roman Hakl, Lucie Ballonova, Barbora Ravcukova, Dita Ricna, Karolina Kozena, Lucie Kratochvilova, Marta Sobotkova, Radana Zachova, Pavel Kuklinek, Pavlina Kralickova, Irena Krcmova, Jana Hanzlikova, Martina Vachova, Olga Krystufkova, Eva Dankova, Milos Jesenak, Martina Novackova, Michal Svoboda, Jiri Litzman, Tomas Freiberger

**Affiliations:** 1 Centre for Cardiovascular Surgery and Transplantation, Brno, Czech Republic; 2 Faculty of Medicine, Masaryk University, Brno, Czech Republic; 3 Faculty of Science, Masaryk University, Brno, Czech Republic; 4 Department of Allergology and Clinical Immunology, St. Anne’s University Hospital in Brno, Brno, Czech Republic; 5 Department of Immunology, 2nd Medical School, Charles University and University Hospital Motol, Prague, Czech Republic; 6 Institute of Clinical Immunology and Allergy, University Hospital Hradec Kralove, Faculty of Medicine in Hradec Kralove, Charles University, Hradec Kralove, Czech Republic; 7 Department of Immunology and Allergology, University Hospital Pilsen, Pilsen, Czech Republic; 8 Department of Immunology and Allergology, Faculty of Medicine in Pilsen, Charles University, Pilsen, Czech Republic; 9 Institute of Rheumatology and Department of Rheumatology, 1st Faculty of Medicine, Charles University, Prague, Czech Republic; 10 Immunia, Prague, Czech Republic; 11 National Centre for Hereditary Angioedema, Department of Pediatrics, Department of Pulmonology and Pathophysiology, Department of Clinical Immunology and Allergology, Comenius University in Bratislava, Jessenius Faculty of Medicine, University Teaching Hospital in Martin, Martin, Slovakia; 12 Institute of Biostatistics and Analyses, Ltd., Brno, Czech Republic

**Keywords:** HAE, C1-INH-HAE, hereditary angioedema, SERPING1, splicing, genotype–phenotype relationship, time to diagnosis

## Abstract

Hereditary angioedema due to C1 inhibitor deficiency (C1-INH-HAE) is a rare and life-threatening condition characterized by recurrent localized edema. We conducted a systematic screening of *SERPING1* defects in a cohort of 207 Czech patients from 85 families with C1-INH-HAE. Our workflow involved a combined strategy of sequencing extended to UTR and deep intronic regions, advanced in silico prediction tools, and mRNA-based functional assays. This approach allowed us to detect a causal variant in all families except one and to identify a total of 56 different variants, including 5 novel variants that are likely to be causal. We further investigated the functional impact of two splicing variants, namely c.550 + 3A > C and c.686-7C > G using minigene assays and RT-PCR mRNA analysis. Notably, our cohort showed a considerably higher proportion of detected splicing variants compared to other central European populations and the LOVD database. Moreover, our findings revealed a significant association between HAE type 1 missense variants and a delayed HAE onset when compared to null variants. We also observed a significant correlation between the presence of the *SERPING1* variant c.-21 T > C in the *trans* position to causal variants and the frequency of attacks per year, disease onset, as well as Clinical severity score. Overall, our study provides new insights into the genetic landscape of C1-INH-HAE in the Czech population, including the identification of novel variants and a better understanding of genotype–phenotype correlations. Our findings also highlight the importance of comprehensive screening strategies and functional analyses in improving the C1-INH-HAE diagnosis and management.

**Supplementary Information:**

The online version contains supplementary material available at 10.1007/s10875-023-01565-w.

## Introduction

Hereditary angioedema (HAE) is a disorder characterized by recurrent bouts of localized subcutaneous or submucosal edema, typically affecting various organs including limbs, intestinal mucosa, genitals, face or airways. These attacks often cause functional damage, severe pain in the abdominal area, breathing obstructions, and overall quality of life is reduced. The most severe manifestation is life-threatening edema of the larynx.

HAE can be classified into three types based on the immunological findings. HAE-1 is characterized by a reduction in both antigenic and functional C1 inhibitor (C1-INH) levels. Patients with HAE-2 have a normal C1-INH protein concentration but impaired C1-INH function. HAE with normal C1 inhibitor (nC1-INH-HAE) primarily arises from defects in the *F12* and *PLG.* Notably, variants in the *F12* gene have been found to predominantly cause HAE in females. Other genes that have been linked to nC1-INH-HAE in few patients include *ANGPT*, *MYOX*, *KNG1*, and *HS3ST6* [1–4].

Both HAE-1 and 2 are inherited in an autosomal dominant mode and are caused by pathogenic variants in *SERPING1*—gene encoding C1-INH and located in the 11q12-q13.1 chromosome. It is composed of eight exons and seven introns. *SERPING1* is a naturally alternatively spliced gene, but the role of alternative transcripts still remains unclear [5]. Whereas pathogenic variants disrupt the C1-INH structure and abolish protein production in HAE-1, variants changing the active center of C1-INH cause normal production levels of dysfunctional protein in HAE-2. C1-INH-HAE prevalence is 1/50,000–1/100,000, without known ethnic differences [6].

C1-INH belongs to the serpin family (serine protease inhibitors), and contributes especially to vascular permeability and inflammation regulation. Edema in HAE-1/2 is the result of an incorrectly regulated contact system in the absence of functional C1-INH and consequent production of bradykinin from kininogen. Bradykinin, as a powerful vasodilator, increases capillary permeability and constricts smooth muscles.

C1-INH levels should theoretically be 50% in dominantly inherited HAE; however, C1-INH serum levels are typically less than 35% of normal [7, 8]. Although the underlying mechanism is not fully understood in most pathogenic variants, the generally assumed cause is haploinsufficiency with an additional negative effect from a defective allele product on the normal allele expression [9].

Interestingly, the HAE severity can range from asymptomatic to very severe, irrespective of the disease-causing variant type, as even the members of the same family carrying the same *SERPING1* alleles have very distinct disease manifestation [10, 11]. It is thus probable that the HAE phenotype is also influenced by some factors other than the causative variant in *SERPING1*. In very rare cases, disease severity was more or less convincingly associated with particular variants or other factors, while no association was demonstrated at all in other cases [11–15].

In this study, we describe the clinical phenotype and genotype of Czech patients with HAE, and provide an overview of *SERPING1* variants identified in Czech HAE patients involving those published previously as well as some novel variants [16–19]. We evaluate their significance and discuss the impact of some previously published variants.

## Material and Methods

### Patients

Two hundred seven patients with HAE from 85 unrelated Czech families were recruited retrospectively for this study through extensive collaboration with clinical immunologists from all over the Czech Republic who treated the patients and collected their data. C1-INH-HAE diagnosis was established based on clinical signs and the following complement measurements: serum C1-inhibitor concentration, C1-inhibitor activity, and C4 level.

### Complement Testing

Over the last 33 years, methods to detect C4 and C1-INH levels have changed in our country. In the 1980s and 1990s, these levels were detected by radial immunodiffusion and later by immunoprecipitation combined with nephelometry or turbidimetry. Since 1996, C1-INH function has been analyzed by the Enzyme-Linked ImmunoSorbent Assay (Quidel MicroVue C1 InhibitorPlus). The normal C1-INH concentration range was 210–390 mg/L, and the normal values for its functional activity were greater than 68% of the reference value for the standard serum.

### Genotyping and Sequencing

DNA was extracted from EDTA-containing whole blood samples using a standard desalting procedure. The variants in *SERPING1* coding regions (exons 2–8) and their adjacent sequences were analyzed using standard Sanger sequencing protocols (primers and conditions available on request). Subsequently, multiplex ligation-dependent probe amplification (MLPA) was performed using the SALSA MLPA P243-A2 SERPING1 kit (MRC-Holland, The Netherlands) to search for large deletions and duplications.

When no variant was detected by either coding region sequencing or MLPA, non-coding regions (3′UTR, 5′UTR, proximal part of intron 6) were amplified and Sanger sequencing of these regions was performed.

All obtained sequences were compared to GenBank reference sequences NM_000062.3 and NP_000053.2. Detected variants’ nomenclature follows Human Genome Variation Society recommendations [20].

### RNA Analysis

Total RNA was extracted from the peripheral blood, PBMCs and HeLa cells. The extracted RNA was reverse transcribed to cDNA with random hexamers. The subsequent PCR was performed in two steps using primers with sequences situated inside exons. Specific reaction conditions and primer sequences were described previously [17, 19]. Amplicons from the second reaction were checked on 2% agarose gels and then characterized by capillary analysis.

### Minigene Assay

Minigene constructs were used to investigate the sequence variant’s effect on RNA splicing. Wild-type and mutant genomic fragments of *SERPING1* comprising appropriate exons and at least 150 bp flanking introns were amplified with primers. PCR products were cloned into multiple cloning sites inside the pET01 vector (MoBiTec). Subsequently, HeLa and/or HepG2 cells (European Collection of Authenticated Cell Cultures) were transfected with the minigene construct. RNA was extracted 24 h after transfection and then RT-PCR was performed. The specific procedure conditions and primer sequences were described previously [17, 19].

### Restriction Analysis

The presence of c.-21 T > C variant in a patient was established by Sanger sequencing of exon 2 or by AvaII restriction analysis of exon 2 amplification products [18]. The variant’s *trans* or *cis* position was determined by analyzing its occurrence among the patient's blood relatives.

### Targeted NGS

Patients’ genomic DNA samples were analyzed firstly on a NextSeq Illumina platform (Illumina, San Diego, CA) using the SureSelect QXT (Agilent Technologies, Santa Clara, CA). The targeted NGS panel comprised exon sequences from genes related to primary immunodeficiencies, including all genes associated with HAE. Intronic sequences of *SERPING1* were also covered by the analysis, except for highly repetitive deep-intronic parts. Library preparation and sequencing were performed according to the manufacturer’s instructions.

Raw data read quality control was performed using the FastQC program [21]. Alignment to the reference hg19 genome was carried out using BWA-MEM [22]. SAMtools was used to sort and index the alignments [23]. The Picard MarkDuplicates tool [24] was employed to mark and remove duplicates. The Vardict program was used to determine genetic variants [25]. Identified variants were annotated with the Annovar tool [26]. Integrative Genomics Viewer (IGV) was employed to visualize read alignment and detected variants [27].

### Databases and Bioinformatics

Interpreting sequence variant impact was based on the criteria established by the American College of Medical Genetics and Genomics (ACMG). Several population and variant databases and bioinformatic tools have been used to annotate variants and estimate variant impact (Supplementary Methods).

## Results

There are 4 major centers specialized in HAE patient treatment in the Czech Republic, and the vast majority of genetic testing has been provided by the Molecular Genetic laboratory CKTCH Brno. Several individual cases were reported to the laboratory by individual specialists as well. Data of the patients were collected over a long time period using available technologies at the time. In 2012, a specialized patient database was introduced providing not only the attending physicians but also the patients with the opportunity to report HAE attacks and disease development.

### Clinical Evaluation of Laboratory Results

Altogether, 207 patients from 85 families were recorded in the Czech Republic. One hundred seventy-five patients from 74 families (87.1%) were diagnosed with HAE-1, and 32 patients from 11 families (12.9%) with HAE-2. The specific data of all patients can be found in Supplementary (Table S1). The data of our cohort are summarized in Table 1.

**Table 1:** Czech HAE patient cohort. (A) Numbers of specific groups of HAE patients. (B) C1-INH and C4 concentration and C1-INH function were crucial to establish diagnosis. The lowest values were considered in case of repeated measurements. C1-INH function and concentration in HAE-1 patients were reduced even in asymptomatic patients, i.e. patients diagnosed before symptom onset, typically blood-related to a proband. There were 9 patients (5%) in the whole cohort that showed normal C1-INH function levels even after repeated testing. Patients with HAE-2 had normal but more often increased C1-INH levels. C4 levels in HAE-1 and HAE-2 symptomatic patients did not differ, with a median 0.05 g/l and 0.06 g/l, respectively. The normal range slightly changed over time, but generally, only 12 out of all 188 (6.4%) patients showed consistently normal C4 levels. Patients with inconclusive complement test results were typically part of families where the variant segregated with the disease and/or was classified unequivocally pathogenic. When the C4 level and C1-INH level and function were considered together, all but one patient (P05505) with available results exhibited at least one abnormal value

(A)	Number		
Patients	207		
Probands	85		
HAE-1 patients	175		
HAE-1 probands	74		
HAE-2 patients	32		
HAE-2 probands	11		
Females	109		
Males	98		
(B)	Median	Range	Typical normal values
Symptomatic HAE-1 patients			
C1-INH concentration (g/l; *n* = 146)	0.06	0.018–0.21	0.210–0.390
C1-INH function (%; *n* = 136)	38	0–78	> 68
C4 concentration (g/l; *n* = 146)	0.05	0.018–0.23	0.100–0.380
Asymptomatic HAE-1 patients			
C1-INH concentration (g/l; *n* = 14)	0.089	0.03–0.168	0.210–0.390
C1-INH function (%; *n* = 13)	56	20–82	> 68
C4 concentration (g/l; *n* = 14)	0.075	0.02–0.11	0.100–0.380
Symptomatic HAE-2 patients			
C1-INH concentration (g/l; *n* = 27)	0.383	0.212–0.765	0.210–0.390
C1-INH function (%; *n* = 27)	45	15–79	> 68
C4 concentration (g/l; *n* = 25)	0.06	0.019–0.21	0.100–0.380
Asymptomatic HAE-2 patients			
C1-INH concentration (g/l; *n* = 3)	0.383	0.35–0.414	0.210–0.390
C1-INH function (%; *n* = 3)	57	33–75	> 68
C4 concentration (g/l; *n* = 3)	0.06	0.05–0.066	0.100–0.380

### Course of the Disease

**Fig. 1:**
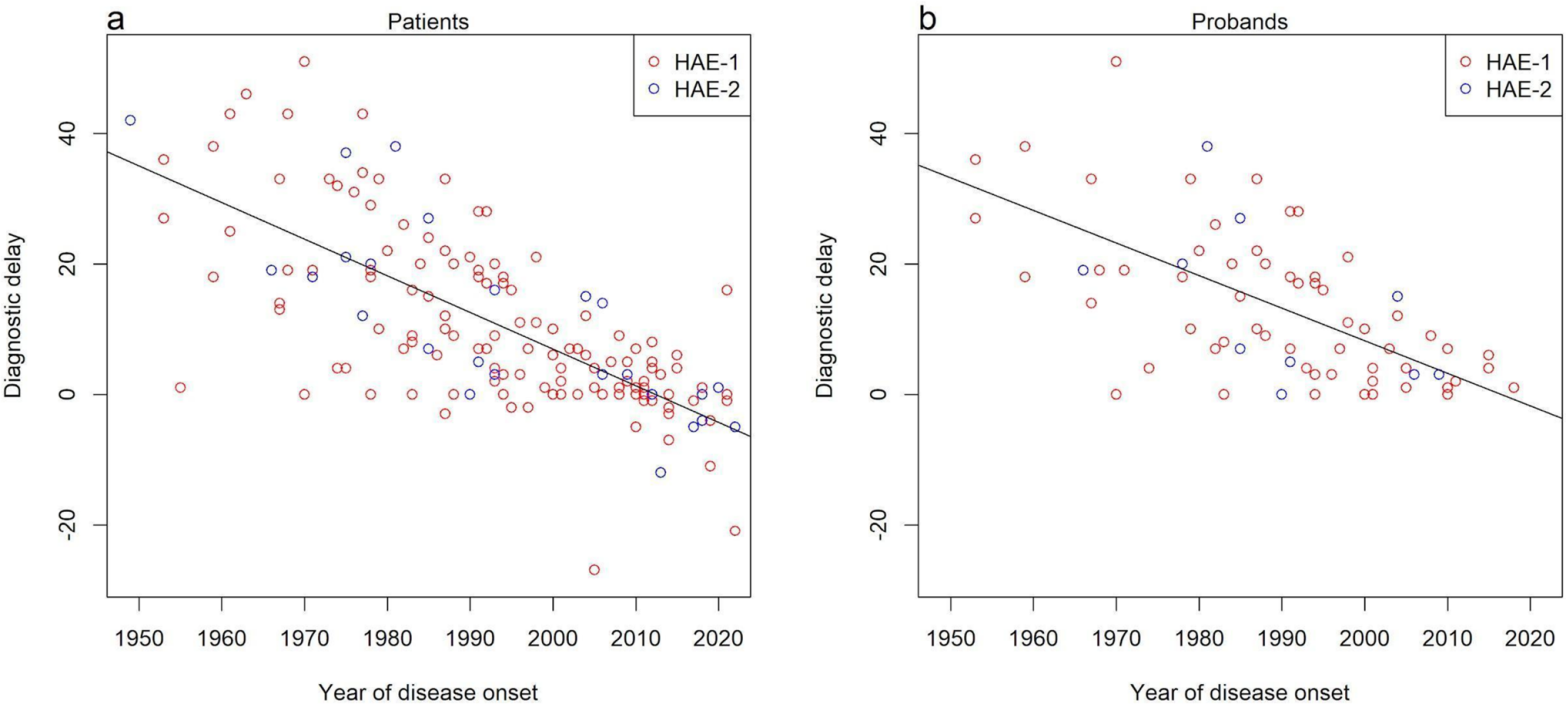
Diagnostic delay, i.e., difference in time between establishing the diagnosis and disease onset. **a** Graph for all Czech patients with available data. **b** Graph for probands of the cohort with available data

The mean age at onset of clinical symptoms was 14 years (range 1–72 years; *n* = 167). Seventy-four patients (44.3%) suffered from their first attack before the onset of puberty (before 13 years of age), and the disease started during puberty (13–16 years of age) in 28 patients (16.8%). A causal variant was detected in 37 patients before the onset of HAE symptoms due to testing HAE patients’ relatives with a known *SERPING1* variant. In 1 patient (P05505 in Table S1), the age of HAE onset was 68 years. Throughout the years, diagnosing HAE has become more achievable with improving immunologic and genetic tests, and the diagnostic delay between the first symptoms and establishing the diagnosis decreased, as shown in Fig. 1.

Information on HAE patient treatment was collected from the Czech national registry of primary immunodeficiencies, where almost all diagnosed HAE patients in the Czech Republic are registered. The data collected between March 2012 and October 2021 were analyzed. A total of 6317 HAE attacks were recorded in 150 patients. Attack location and their treatment are specified in Tables S2 and S3, respectively. Long-term prophylaxis was used in 95 patients.

We calculated the clinical severity score for each patient in our cohort using available information on age of onset, attack location, and long-term prophylaxis usage, following the method introduced by Bygum et al. [28]. It should be noted that, except for age of onset, the score calculation was based solely on the Czech national registry of primary immunodeficiencies data, capturing information from 2012 till 2021. However, the score considers swelling occurrences at any point in a patient’s lifetime, which would potentially result in higher scores in some patients when calculated based on their complete “lifetime” records.

### Genetic Analysis

Several methods were used to find causative sequence defects in *SERPING1* in HAE patients. When the HAE genetic diagnostics were introduced, denaturing gradient gel electrophoresis was used to search for a defect in *SERPING1* coding parts, followed by Sanger sequencing of regions showing a pattern that differed from the reference control. Later, direct Sanger sequencing of all coding parts and adjacent intronic sequences of the gene was performed. If the causal variant was not found in a patient, MLPA was used to search for large defects in the gene. Nonetheless, causative sequence variants in the gene were still not found in some cases. Then, we tried to gain RNA from blood samples of the patients and their relatives—both healthy and suffering from HAE. To uncover potential splicing defects, we used their cDNA to amplify several *SERPING1* mRNA segments overlapping particular exon boundaries, and search for exon inclusion abnormalities using fragment analysis [17].

Generally, we tried to establish and confirm the intronic and splicing variants’ impact independently. Typically, we applied minigene assay to investigate the effect of the detected sequence variant on RNA splicing, as described in the “Material and Methods” section [19].

The workflow of methods currently used to detect and evaluate causal variants by our laboratory is illustrated in Fig. S1. Using this set of methods, we detected a sequence variant that we considered as causative or probably causative in 206 out of 207 in our cohort of Czech patients, i.e. in 84 families out of 85. We found 56 unique pathogenic or likely pathogenic sequence variants.

These variants included 18 different missense, 4 nonsense, 13 frameshift, 16 splicing variants, and 5 copy number variations (CNVs) (Tables 2, 3, 4, 5, and 6).

**Table 2:** Missense variants found in the Czech cohort. Variants were evaluated by in silico tools (CADD, Polyphen, and SIFT). The Proof of Pathogenicity column provides information on which the variant evaluation is based. 'MP' indicates multiple published patients (including this study), while 'FP' signifies functional proof of variant impact with referenced articles containing such evidence. The *Proof of pathogenicity* column provides information on which the variant evaluation is based with “MP” indicating multiple published patients (including this study), and “FP” functional proof of variant impact. The resources are indicated in the *References* column, and articles containing functional proof are [33, 34, 57]$$$$$$$$$. The numbers of patients and probands of our cohort are given in corresponding columns. ^#^ indicates previously published probands/patients, and x in ^x#^ indicates the number of them. Two substitutions in the position c.550 were included in the splicing variants subset (Table 5) because their pathomechanism is primarily disruption of mRNA splicing

Variant cDNA	Variant protein	CADD	Polyphen category	SIFT category	Proof of pathogenicity	ACMG evaluation	Number of probands	Number of patients	References
c.498C > A	p.Asn166Lys	24	Probably damaging	Damaging	MP	Pathogenic	1	1	[12, 29–32]
c.503C > A	p.Ala168Asp	22.9	Probably damaging	Damaging	MP, FP [33, 34]	Pathogenic	1	3	[30, 31, 33, 34]
c.506 T > C	p.Phe169Ser	29	Probably damaging	Damaging	MP	Pathogenic	1	2	[35, 36]
c.548 T > C	p.Leu183Pro	32	Probably damaging	Damaging	MP	Pathogenic	1^#^	1^#^	[17, 37, 38]
c.614G > A	p.Cys205Tyr	24.3	Benign	Damaging	MP	Pathogenic	1	3	[37, 39, 40]
c.629 T > C	p.Leu210Pro	24.8	Probably damaging	Damaging	MP	Pathogenic	1^#^	2^1#^	[17, 38, 41]
c.706 T > G	p.Phe236Val	24.8	Probably damaging	Damaging		Likely pathogenic	1^#^	2^1#^	[17]
c.722G > C	p.Arg241Pro	14.82	Probably damaging	Tolerated	MP, FP [33]	Likely pathogenic	1	2	[33, 36]
c.743C > G	p.Pro248Arg	23.2	Probably damaging	Damaging	MP	Likely pathogenic	1	3	[36, 42]
c.793 T > G	p.Trp265Gly	25.4	Probably damaging	Damaging		Likely pathogenic	1^#^	3^1#^	[17]
c.1046 T > C	p.Leu349Pro	26.8	Probably damaging	Damaging	MP	Pathogenic	1^#^	2^1#^	[17, 43]
c.1195C > T	p.Pro399Ser	23.2	Probably damaging	Damaging	MP	Pathogenic	1	2	[36, 38, 44, 45]
c.1202 T > A	p.Ile401Asn	25.5	Probably damaging	Damaging	MP	Likely pathogenic	1^#^	2^1#^	[17, 46]
c.1322 T > A	p.Met441Lys	25.1	Probably damaging	Damaging		Likely pathogenic	1^#^	3^1#^	[17]
c.1346 T > C	p.Leu449Pro	28.9	Probably damaging	Damaging	MP	Pathogenic	1	1	[30, 47]
c.1361 T > G	p.Val454Gly	28.2	Probably damaging	Damaging	MP	Pathogenic	4^1#^	8^1#^	[17]
c.1396C > T	p.Arg466Cys	25.3	Probably damaging	Damaging	MP, FP [57]	Pathogenic	5^2#^	15^5#^	[30, 32, 34, 36, 38, 45, 48–60]
c.1397G > A	p.Arg466His	23.4	Benign	Damaging	MP, FP [57]	Pathogenic	6^2#^	17^4#^	[28, 30, 32, 39, 46, 48, 50, 52, 54, 57, 61–64]

**Table 3:** Nonsense variants found in the Czech cohort. The *Proof of pathogenicity* column provides information on which the variant evaluation is based with “null” indicating that the variant probably results in no gene product and “MP” indicates multiple published patients (including this study). The resources are indicated in the *References* column. Numbers of patients and probands of our cohort are given in corresponding columns. ^#^ indicates previously published probands/patients of our cohort and x in ^x#^ indicates the number of them

Variant cDNA	Variant protein	Proof of pathogenicity	ACMG evaluation	Number of probands	Number of patients	References
c.209C > G	p.(Ser70*)	Null	Pathogenic	1^#^	1^#^	[18]
c.897G > A	p.(Trp299*)	Null, MP	Pathogenic	1^#^	3^1#^	[17, 30, 32]
c.1036C > T	p.(Gln346*)	Null, MP	Pathogenic	1	3	[30, 35, 36]
c.1420C > T	p.(Gln474*)	Null, MP	Pathogenic	1	3	[12, 30, 56]

**Table 4:** Frameshift variants found in the Czech cohort. The column *Proof of pathogenicity* provides information on which the variant evaluation is based—“null” indicating that the variant probably results in no gene product, 'MP' indicates multiple published patients (including this study), while 'FP' signifies functional proof of variant impact with referenced articles containing such evidence. The resources are indicated in the *References* column, and articles containing functional proof are [17]. Numbers of patients and probands of our cohort are given in corresponding columns. ^#^ indicates previously published probands/patients of our cohort, and x in ^x#^ indicates the number of them. The variant c.726_777del included in this table comprises more than 20 bases but because it does not affect the whole exon, it was not included in the CNV subset (Table 6). On the other hand, the deletion c.1225_1249 + 19del was placed in the splicing variant table (Table 5) as it primarily disrupts mRNA splicing

Variant cDNA	Variant protein	Proof of pathogenicity	ACMG evaluation	Number of probands	Number of patients	References
c.120_121del	p.(Gly41Argfs*16)	Null, MP, FP [17]	Pathogenic	1^#^	3^#^	[17, 18, 33, 36, 42, 47, 56]
c.151_152del	p.(Ser51Glnfs*6)	Null	Pathogenic	1	2	novel
c.160del	p.(Leu54Tyrfs*25)	Null	Pathogenic	1^#^	3^1#^	[18]
c.305_317del	p.(Pro102Leufs*42)	Null, FP [17]	Pathogenic	2^1#^	14^1#^	[17]
c.600dup	p.(Lys201Glnfs*56)	Null, MP	Pathogenic	1^#^	2^1#^	[12, 30, 36, 47, 64]
c.650del	p.(Gly217fs*15)	Null, MP	Pathogenic	1^#^	1^#^	[17, 53]
c.726_777del	p.(Leu243Serfs*19)	Null	Pathogenic	1	7	novel
c.795_796delGGinsT	p.(Trp265Cysfs*14)	Null	Pathogenic	1	2	novel
c.855_856del	p.(Arg286Profs*18)	Null	Pathogenic	1^#^	2^#^	[18]
c.1115del	p.(Gln372Argfs*25)	Null	Pathogenic	1^#^	2^1#^	[17]
c.1283del	p.(Cys428Leufs*3)	Null, MP	Pathogenic	1^#^	1^#^	[17, 33]
c.1284_1285del	p.(Cys428Trpfs*44)	Null, MP	Pathogenic	3^1#^	4^1#^	[18]
c.1460_1466del	p.(Lys487Metfs*87)	Null	Pathogenic	1	5	novel

**Table 5:** Splicing variants found in the Czech cohort. The Proof of Pathogenicity column provides information on which the variant evaluation is based. 'MP' indicates multiple published patients (including this study), while 'FP' signifies functional proof of variant impact with referenced articles containing such evidence. The resources are indicated in the *References* column, articles containing functional proof are [17, 19, 35, 66, 73]. Numbers of patients and probands of our cohort are given in corresponding columns. ^#^ indicates previously published probands/patients of our cohort, and x in ^x#^ indicates the number of them. Two substitutions in the position c.550 and the deletion c.1225_1249 + 19del were included in this table because their pathomechanism is primarily disruption of mRNA splicing

Variant cDNA	Intron	Proof of pathogenicity	ACMG evaluation	Number of probands	Number of patients	References
c.-22-19_-22-4del	1	MP	Likely pathogenic	1	2	[36, 65]
c.51 + 5G > A	2	MP, FP [35, 66]	Pathogenic	1	1	[30, 31, 35, 66]
c.550G > A	exon 3	MP, FP [17]	Pathogenic	1^#^	1^#^	[17, 28, 31, 32, 35, 36, 38–40, 42, 45, 50, 51, 56, 58, 62, 67–70]
c.550G > T	exon 3	MP, FP [17]	Pathogenic	1^#^	1^#^	[17, 36, 56]
c.550 + 3A > C	3	FP [this study]	Likely pathogenic	1	1	novel
c.551-2A > G	3	MP, FP [17]	Pathogenic	2^1#^	11^1#^	[17, 36, 49, 62]
c.685 + 1del	4	FP [17]	Pathogenic	1^#^	4^1#^	[17]
c.685 + 2_685 + 13del	4	FP [17]	Pathogenic	1^#^	4^1#^	[17]
c.686-12A > G	4	MP, FP [17]	Pathogenic	1^#^	1^#^	[17, 44, 47, 55]
c.686-7C > G	4	MP, FP [this study]	Pathogenic	1	2	[36]
c.686-1G > T	4	MP	Pathogenic	1	1	[38]
c.1029 + 384A > G	6	MP, FP [19]	Pathogenic	3^1#^	15^12#^	[19, 34, 71, 72]
c.1225_1249 + 19del	7	FP [17]	Pathogenic	1^#^	2^1#^	[17]
c.1249 + 1G > A	7	MP	Pathogenic	1	1	[37, 43, 49, 61]
c.1249 + 2 T > C	7	MP	Pathogenic	1	1	[56]
c.1249 + 5G > A	7	MP, FP [17, 73]	Pathogenic	1^#^	1^#^	[17, 36, 65, 73]

**Table 6:** Large deletions and duplication found in the Czech cohort. Numbers of patients and probands of our cohort are given in corresponding columns. The variant c.726_777del was included in the frameshift set (Table 4) even though it comprises more than 20 bases because the deletion does not affect a whole exon

Variant cDNA	Number of probands	Number of patients
EX1-6del	1	3
EX1-8del	2	2
EX4del	9	15
EX7del	2	6
EX5-6dup	1	1

#### Missense Variants

**Fig. 2:**
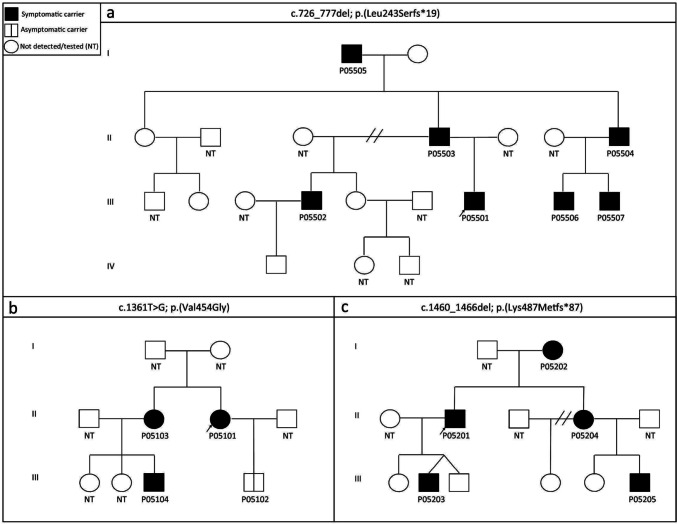
Family trees of HAE patients. HAE-affected family members carrying the causal variant are shown in black, Asymptomatic/presymptomatic carriers of causal variants are depicted by a partitioned symbol, and healthy individuals are depicted by a blank symbol. Individuals who were not tested are signified by NT. **a** The variant c.726_777del was identified in 7 members of one family with HAE-1. The variant was first revealed in a 10-year-old boy (P05501) and his father, who both showed relatively severe HAE symptoms, and their condition started at a young age, at 9 and 7 years, respectively. Further genetic analysis revealed the same variant in another 4 members of the family. Interestingly, the oldest member of the family—an 81-year-old grandfather (P05505) of this 10-year-old boy—suffered from only 2 attacks in his life, both appearing before establishing his diagnosis perioperatively at 68 and 73 years of age. His C1-INH and C4 levels, and C1-INH function were normal. All other members with the detected variant had low C4 and C1-INH levels as well as C1-INH function, and all of them also showed HAE symptoms. 4 asymptomatic members with normal C4 and C1-INH levels were tested, and the variant was not detected in their DNA samples. **b** The variant p.Val454Gly was among other patients also found in 4 members of the depicted family. Three family members (P05101, P05103 and P05104) suffered from HAE attacks and also showed laboratory HAE symptoms, whereas 29-year-old P05102 showed only C1-INH concentration and function deficit with no clinical symptoms of HAE. **c** Seven base pair deletion c.1460_1466del was detected in a family with 5 patients. The HAE phenotype in this family segregates with the presence of the variant and the disease course is quite severe in all affected members—in P05205, the attacks appeared as early as 2 years of age. The variant was not found in 4 other asymptomatic family members tested

Eighteen different missense variants were detected in 30 probands which accounted for 35.3% of all probands (Table 2). The most prevalent variants, p.Arg466His (17 patients in 6 families) and p.Arg466Cys (15 patients in 5 families) in exon 8, were connected to HAE-2 phenotype. Interestingly, the most common missense variant causing HAE-1, p.Val454Gly (8 patients in 4 families), was also located in exon 8. The potential effect of this missense variation was estimated by three different prediction programs, all of them predicting the change to have damaging effects on the protein (Table 2). It has been previously described only once, also in a patient of Czech origin [17]. Now we report the variant in another three probands. In one of the families, the variant was detected in the affected father (P05601) and also in his daughter (P05602) when she was 10 years old. She had not had any HAE attacks but showed low C1-INH concentration and function. It was also found in another family depicted in Fig. 2b.

All other detected missense variants were specific to particular families, although they had been described previously in HAE patients (see references in Table 2). The potential impacts of all these variants were estimated by in silico tools and the variants were evaluated based on ACMG rules. Specific concern was paid to functional studies, which, regrettably, have been published for only four variants to date, and to the number of previously described HAE patients carrying the respective variant (Table 2).

Needless to add, two other substitutions at the c.550 position were detected, but as these variants’ pathomechanism is de facto mRNA splicing disruption [17, 42], they were included in the splicing variant subset.

#### Nonsense Variants

Four different nonsense variants were identified in 10 patients from 4 families and they comprised 4.7% of all probands. All these variants had been described before as causative for HAE-1 (Table 3).

#### Frameshift Variants

Detected frameshift variants comprised 11 deletions, 1 duplication, and 1 indel variant. Altogether, they were detected in 48 patients from 16 families and accounted for 18.8% of probands (Table 4).

A novel 2-base deletion, c.151_152del, was identified in exon 3 in a mother with HAE symptoms (P03501) and her infant daughter (P03502). The variant potentially leads to the frameshift and premature stop codon introduction (p.(Ser51Glnfs*6)) in the mRNA. Both the mother and her daughter carrying this variant did not have any other rare *SERPING1* variation. They displayed HAE symptoms, and their complement measurements showed a deficient C1-INH level and function as well as below-normal C4 level.

Another novel deletion, c.726_777del; p.(Leu243Serfs*19), was identified in 7 members of one family with HAE-1 (P05501- P05507; Fig. 2a).

In a family with 5 patients (P05201-P05205), we additionally detected 7 base deletion c.1460_1466del; p.(Lys487Metfs*87) in exon 8 (Fig. 2c), which had not been described before.

Furthermore, we found a novel indel variant leading to frameshift c.795_796delGGinsT; p.(Trp265Cysfs*14) in a patient (P05301) and her daughter (P05302) both showing clinical and laboratory signs of HAE.

Some of the detected deletions comprise more than 20 bases [74] and therefore should fall rather into the gross deletion category. However, as they do not affect the whole exon(s) and their pathological consequences are frameshift and introduction of a premature stop codon, we included them in the frameshift category.

To categorize the deletion c.1225_1249 + 19del, the situation is even more complicated because the variant causes primarily splicing defects [17] and was therefore included in the splicing variant subset.

#### Large Deletions and Duplications

A major part of gross variants has been detected by MLPA; however, two deletions mentioned in the previous paragraph, which technically should be gross deletions, were detected by Sanger sequencing. When these are not taken into account, the other gross deletions of one or several exons were detected in 26 patients from 14 families, which comprise 16.5% of the cohort. A duplication of exons 5–6 was detected in 1 patient (1.2% of the probands).

#### Splicing Variants

As mentioned before, 2 missense and 1 frameshift variant found in our cohort disrupt mRNA splicing. In addition, we found 13 other splicing variants. Thus, in total, we detected splicing variants in 49 patients from 19 families which account for 22.4% of probands.

Out of the 16 detected variants, 7 disrupted canonical splice site positions, which is a well-established pathogenic mechanism, and additionally, functional studies have been described for 4 of these variants [17]. In our cohort, we identified 9 variants located in non-canonical splice site positions, with 8 of them being previously published. Functional studies have been conducted for 6 of these variants (Table 5). In this study, we performed functional studies on a previously published variant as well as a novel variant that we detected.

**Fig. 3:**
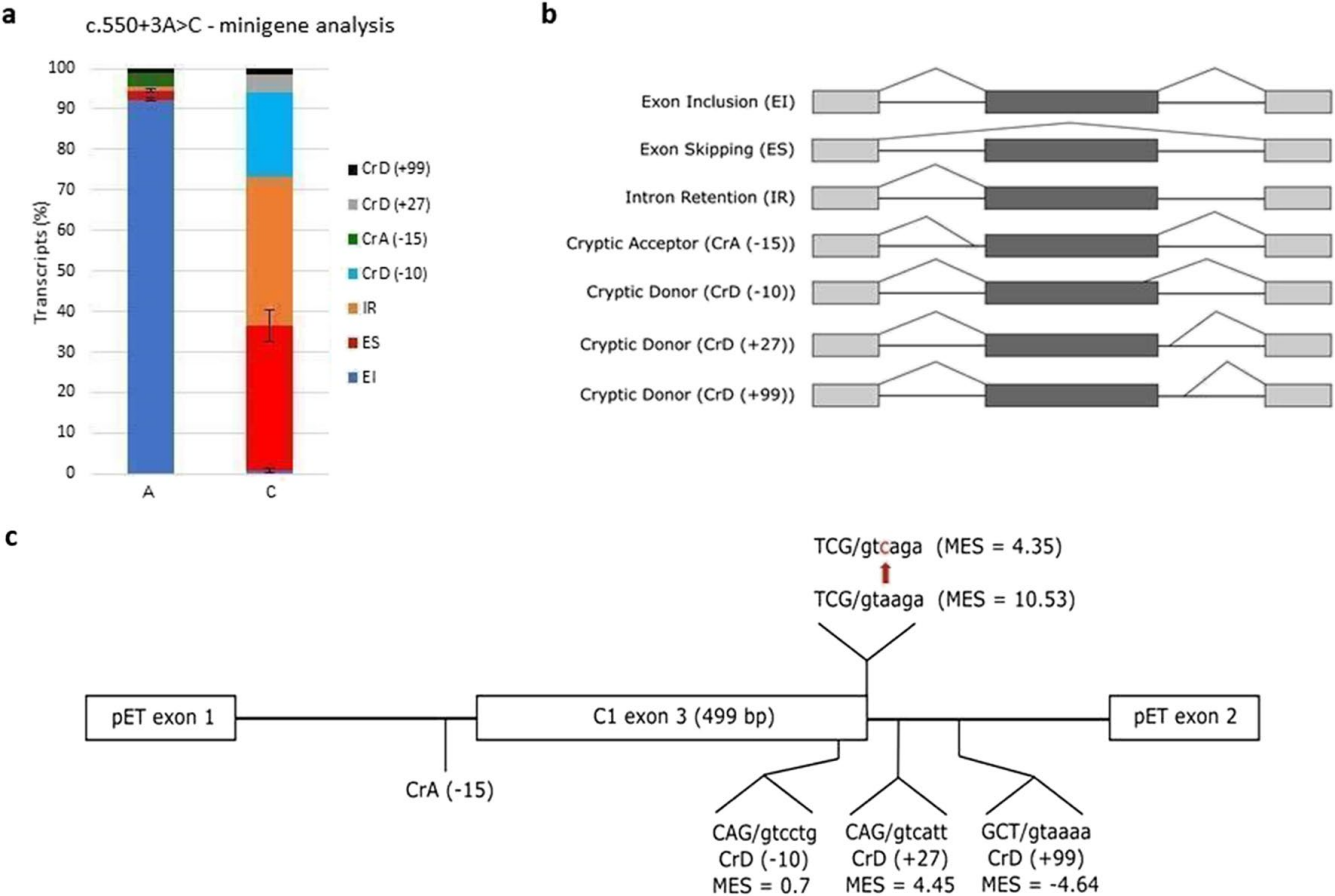
Minigene splicing analysis of a novel variant c.550 + 3A > C. The wild-type and mutant genomic fragments of *SERPING1* comprising exon 3 and flanking upstream (229 bp) and downstream (255 bp) intron sequences, were cloned into pET01 vector and HepG2 cells were transfected with these minigenes. Capillary electrophoresis of RT-PCR products showed aberrant splicing in nearly 100% of mutated minigene construct transcripts. Several different aberrant transcripts were detected. The most abundant was intron 3 retention followed by exon 3 skipping. Transcripts using cryptic donor splice sites − 10 and + 27 were found in the mutant minigene analysis, whereas they were not detected in the wild type at all. **a** Minigene analysis results: column A shows transcript proportions resulting from control minigene construct representing c.550 + 3A; column C shows transcript proportions resulting from minigene construct representing c.550 + 3C. **b** Scheme of the transcripts detected in the minigene analysis. **c** Scheme of the pET minigene construct. Cryptic splice sites found in the analysis and their exact sequences are displayed. MaxEnt Score values (MES) are specified beneath each splice site

The impact of the substitutions in exon 3’s last nucleotide (position c.550) on splicing was previously functionally evaluated using RNA analysis and was established as pathogenic [75]. In the same splice site, we detected a novel variant, c.550 + 3A > C, in a patient P02201 and as the variant had not been previously reported, we tried to functionally evaluate it. Unfortunately, none of the patient’s family members were available for testing. The variant potentially affecting the donor splice site (5'ss) of exon 3 was analyzed in silico by MaxEnt Score [76], and the ratio between mutated and wild-type 5'ss sequence was 0.41—a number suggesting a substantial effect on splicing. According to Le Guédard-Méreuze et al. [77], substitution + 3A > C is prone to cause a splicing defect even if the donor splice site does not contain any further nucleotide changes. We performed minigene analysis (detailed procedure described in Supplement Methods) to confirm the deleterious effect on splicing and it showed aberrant splicing in nearly 100% of mutated minigene construct transcripts (Fig. 3).

**Fig. 4:**
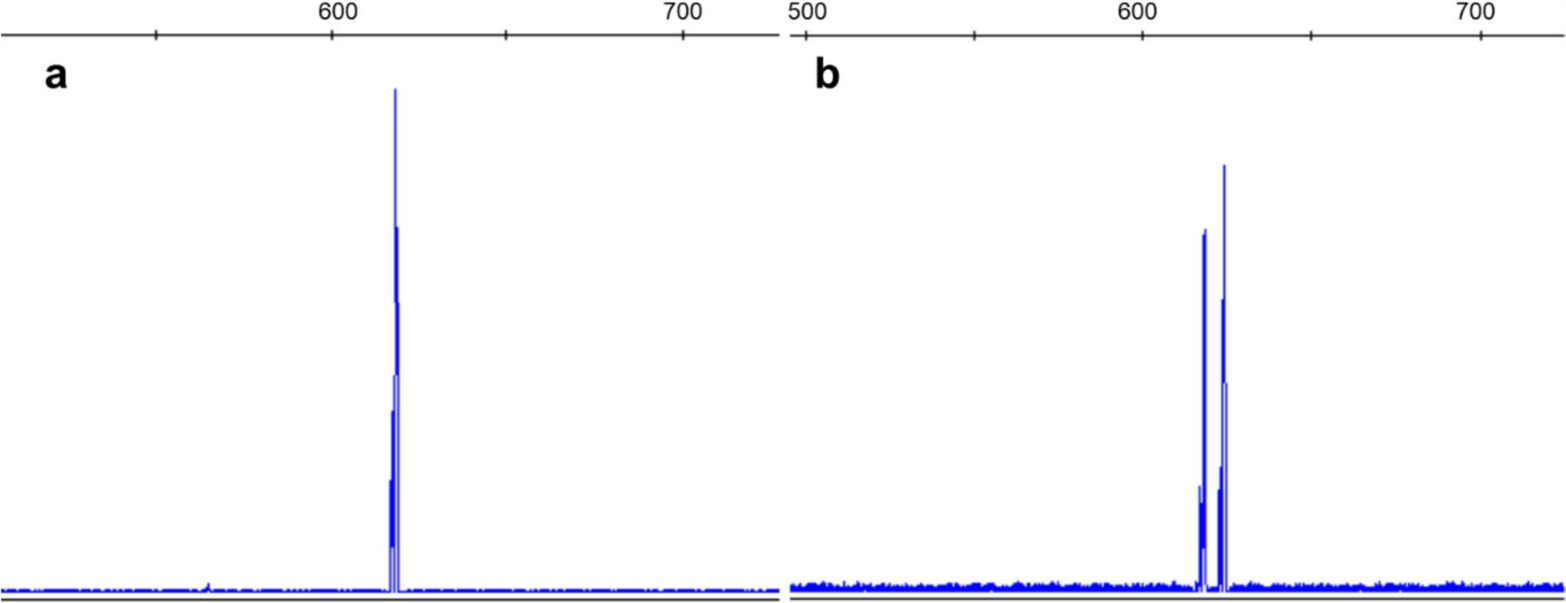
mRNA analysis of patients carrying variant c.686-7C > G. Capillary electrophoresis was performed on PCR products amplified by primers annealing to exons 4 and 7. **a** Healthy control shows only one wild type peak. **b** Both patient sample results show 2 peaks—reference transcript peak and a peak corresponding to a 6 bp longer transcript using a de novo created acceptor splice site. Aberrant and normal transcript proportion was roughly equal, which corresponds to complete impairment caused by the variant. The variant preserves a reading frame and leads to incorporation of additional two amino acids (proline-alanine) into the polypeptide chain

Variant c.686-7C > G was detected in a mother and her daughter (P03001-P03002), both affected with HAE symptoms. This variant had been described before [7], but its impact had not been yet fully evaluated. Therefore, we extracted RNA from both patients’ blood and analyzed samples by RT-PCR and fragment analysis (Supplementary Methods), which showed complete impairment caused by the variant (Fig. 4).

### Variant Type and Course of Disease

**Fig. 5:**
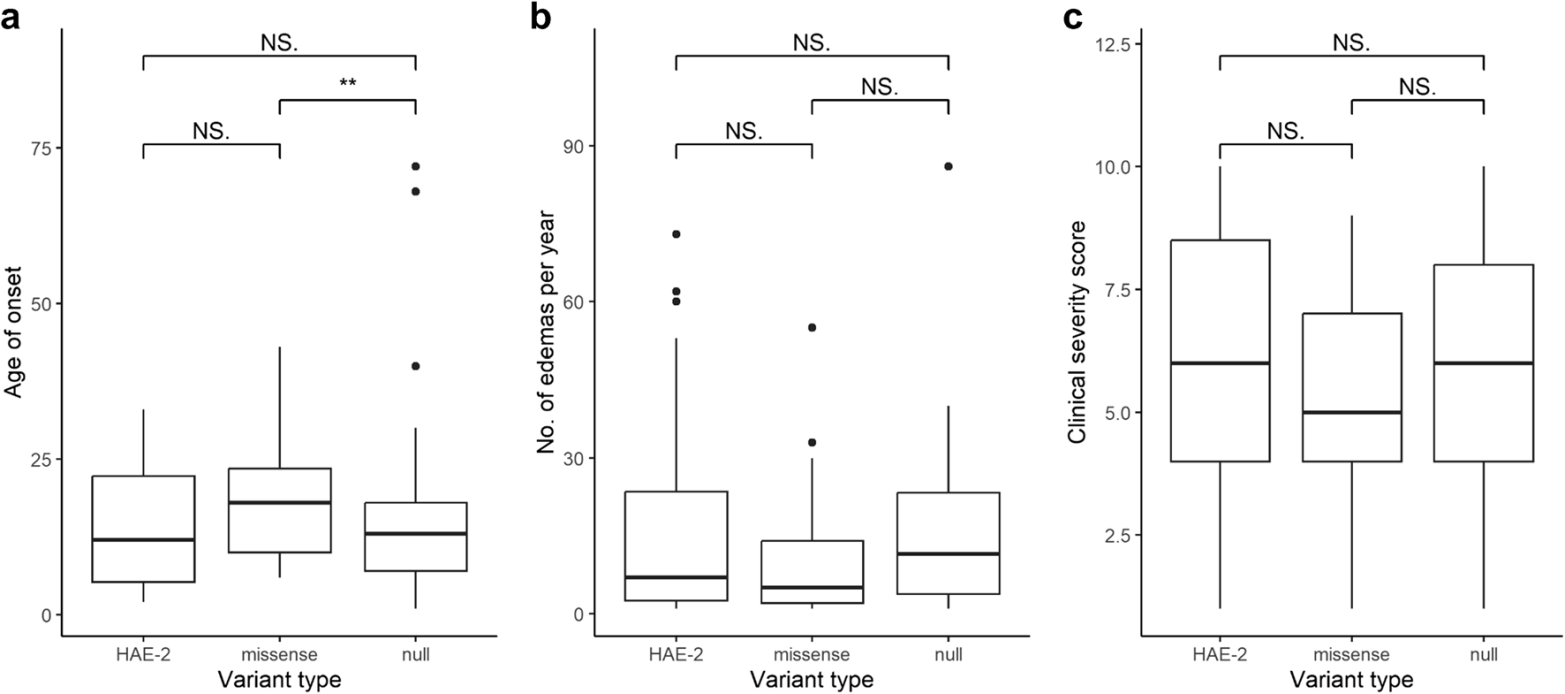
Impact of causal variant types on HAE phenotype. HAE Patients were classified into different groups based on their specific causal variant types: HAE-2 variants causing damage to the active center of *SERPING1*, other missense variants, and null variants preventing C1 inhibitor formation. The primary objective was to investigate the impact of these variant types on various phenotypic characteristics. **a** The impact of causal variant type on age of HAE onset was analyzed in 26, 32, and 109 patients from HAE-2, missense and null groups, respectively. Analysis showed no significant association in relation to the HAE-2 group. However, null variants in patients were significantly associated with lower age of HAE onset compared to those with the missense variant (Kruskal–Wallis; *p* = 0.023). **b** The impact of causal variant type on HAE attack frequency was analyzed in 27, 34, and 108 patients from HAE-2, missense, and null groups, respectively. Analysis revealed no significant associations. **c** The impact of causal variant type on clinical severity score [28] was analyzed in 20, 31, and 79 patients from HAE-2, missense, and null groups, respectively. No significant association was found

We classified patients based on their genetic variants. Those with variants that prevented the production of a functional transcript were grouped as null variants, while those with a missense defect in Arg466 were classified as HAE-2. Patients with other missense variants were categorized as missense. We then studied the patient groups to determine potential connections between the type of causal variant with the age of onset, the number of disease attacks per year, and the clinical severity score. Although there was no apparent association between the type of causal variant and the frequency of HAE attacks among the groups or clinical severity score, patients with missense variants showed a significantly higher age of HAE onset compared to those with null variants (Kruskal–Wallis; *p* = 0.023; Fig. 5).

### Potential Impact of c.-21 T > C on HAE Course

**Fig. 6:**
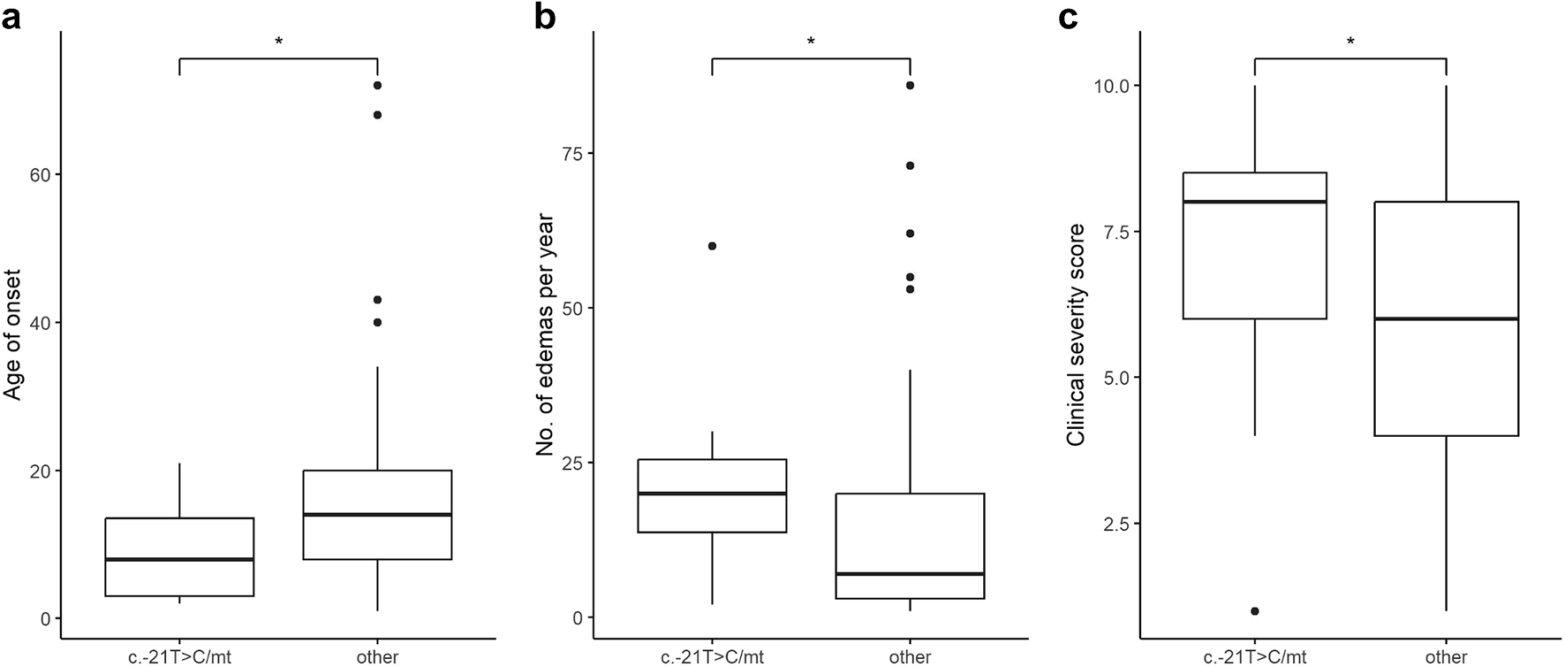
Influence of the variant c.-21 T > C in *trans* conformation with the disease-causing variant on disease phenotype. In the analysis, two groups were compared: patients carrying the c.-21 T > C variant in *trans* conformation with causal variant, depicted in the graph as c.-21 T > C/mt, and patients who did not carry the c.-21 T > C variant or had this variant in *cis* form, depicted as other **a** The impact on the age of HAE onset was evaluated by comparing 12 patients carrying c.-21 T > C in *trans* conformation with causal variant, 148 patients that did not carry c.-21 T > C or have this variant in *cis* form. The presence of the variant c.-21 T > C in *trans* conformation was significantly associated with a lower age of HAE onset (Mann–Whitney; *p* = 0.024). To determine how much this result is affected by the type of causal mutation, we determined the ratio of causal mutation types in the both groups: in the group carrying the c.-21C > T variant the ratio was 1:1:4 and in the other 1:1.1:4.1 (HAE-2:missense:null). **b** The impact on the HAE attack frequency was evaluated by comparing 12 patients carrying c.-21 T > C in *trans* conformation with 150 patients that did not carry c.-21 T > C or have this variant in *cis* form. The presence of the variant c.-21 T > C in *trans* conformation was significantly associated with a higher number of attacks per year (Mann–Whitney; *p* = 0.018). **c** The association of c.-21 T > C in *trans* conformation with Clinical severity score was evaluated by comparing 11 patients carrying c.-21 T > C in *trans* conformation with 134 patients that did not carry c.-21 T > C or have this variant in *cis* form. The presence of the variant c.-21 T > C in *trans* conformation was significantly associated with a higher Clinical severity score (Mann–Whitney; *p* = 0.045)

We examined the patients for the presence of the exonic variant c.-21 T > C in *trans* conformation with the disease-causing variant. It was possible to unambiguously determine c.-21 T > C in *trans* form in 12 patients in our cohort. Its presence was significantly associated with a lower age of HAE onset (Mann–Whitney; *p* = 0.024; Fig. 6a), a higher number of attacks per year (Mann–Whitney; *p* = 0.018; Fig. 6b), and a higher clinical severity score (Mann–Whitney; *p* = 0.048; Fig. 6c).

## Discussion

Here, we present a report of clinical and genetic data from Czech C1-INH-HAE patients (Table S1), which is an update on the whole historical cohort diagnosed in the past years, including previously published cases [16–19].

### Fast and Precise

As we have shown, it took decades to come to conclusive genetic diagnosis in some families [19], but with the advancement of molecular biological techniques, the diagnosis can be reached much faster, with a notable increase in sensitivity. Earlier single-center observations [16] were confirmed in a larger number of individuals from all over the country, as shown on Fig. 1. The time between the first attack of the disease and establishing the diagnosis diminished during the years. Reaching a conclusive diagnosis in the first occurrence in a family of course presents a much more demanding task than when investigating family members, but as seen in Fig. 1b, the diagnostic delay substantially decreased, even in probands. Based on current guidelines, genetic testing is not necessary to establish HAE diagnosis [78]. However, as C1-INH levels and activity, and C4 levels tend to vary between attacks and remissions, it might be essential to identify the disease-causing variant in a patient when C1-INH-HAE is suspected, but the complement test results are inconclusive. It is therefore favorable to confirm the disease genetically in young children where interpreting the complement test results might be especially tricky, and first HAE symptoms could easily be misinterpreted. Also, thanks to genetic counseling and testing for a familial variant the diagnosis can be established in relatives before symptoms emerge, which might prevent them from life-threatening manifestations. Interestingly, one patient in our cohort had a very mild course of disease – the first attack appeared perioperatively at 68 years of age (P05505) and the patient also exhibited normal C4 level, as well as C1-INH level and function. This attack might have not been recognized as an HAE incident, were he not a member of a large family of HAE patients with a formerly established diagnosis.

Only one patient (P01701) from our cohort remained undiagnosed after performing all advanced molecular testing. However, we were able to detect variants classified as pathogenic or potentially pathogenic based on ACMG criteria in all other Czech patients.

Incorporating NGS into the detection method spectrum might be quite useful, specifically, when targeted to intronic and UTR *SERPING1* regions and to other previously described genes related to HAE phenotypes. Recently, we also validated targeted NGS to detect large deletions and duplications, and we are able to search for gross rearrangements and intronic/UTR variants in one step.

### Variant Spectrum

The diversity of the identified pathogenic or probably pathogenic variants in Czech patients (Fig. S2) confirmed the heterogeneity of causal variants observed in other countries [44, 47–49].

**Fig. 7:**
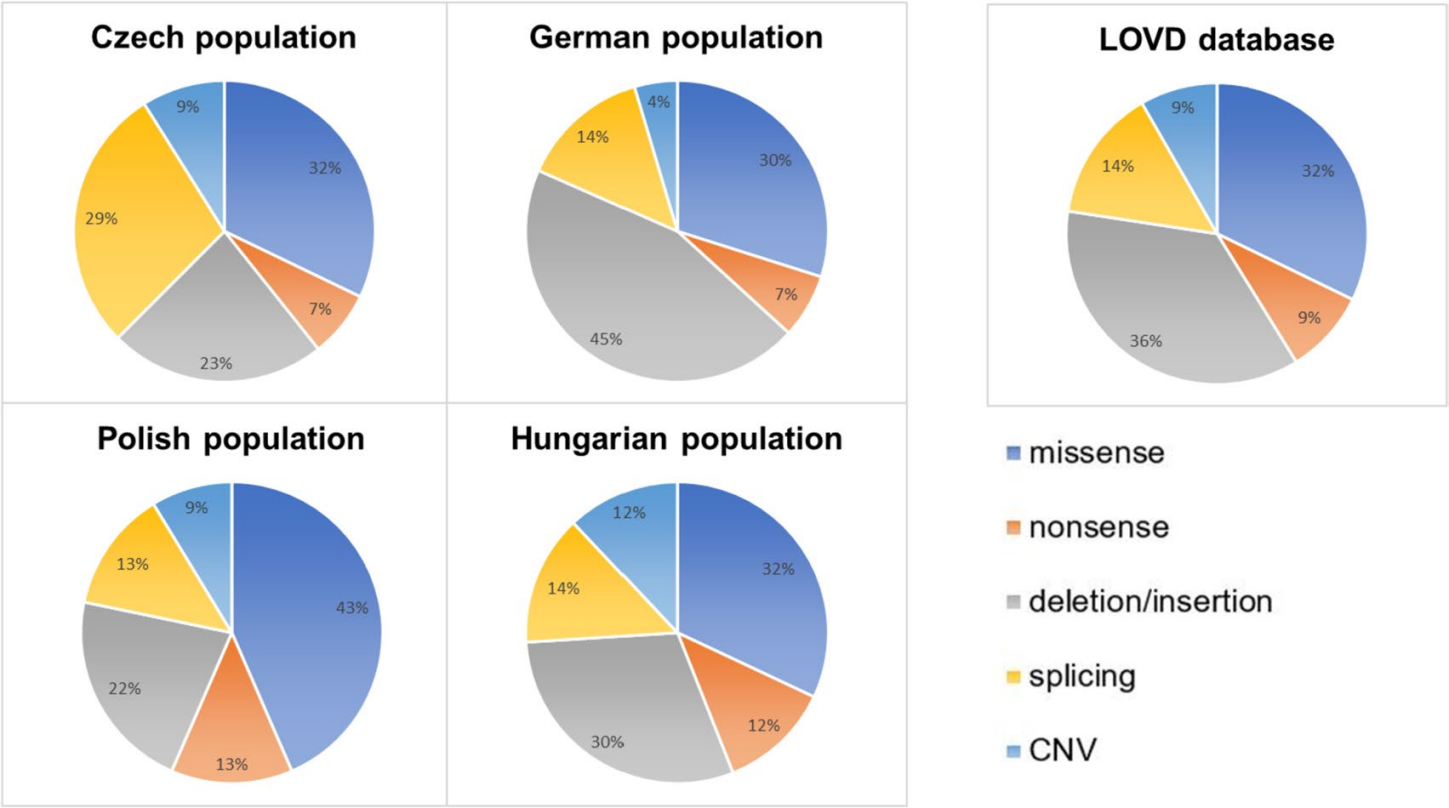
Comparison of HAE causal variant types distribution in Czech cohort, other central European populations, and LOVD worldwide dataset. The data for the Polish population were taken from Obtulowicz et al. [65], and LOVD, while the data for the Hungarian population were taken from Szabó et al. [34]. The data for the German population were taken from LOVD. Regrettably, data for Slovak and Austrian populations, which should be closest to the Czech population from a historical point of view, are not available. The analysis shows that most types of variants were present in similar amounts in all populations studied. However, the proportion of causal splicing variants was remarkably higher in Czech patients than in the other populations and the LOVD dataset

When comparing the proportion of various detected variant types in our cohort with the worldwide dataset (LOVD database [9, 76]), most variant types are of similar amounts. Only in our dataset, the proportion of causal splicing variants is remarkably higher (Fig. 7). This may be due to the higher prevalence of splicing defects in Czech patients, but also because our group focuses specifically on splicing analysis. It might seem to be a result of our specific approach to variant classification; however, the same approach was applied also by Drouet et al. [9] who reviewed data on pathogenic/likely pathogenic *SERPING1* variants from the LOVD database, which we compared our data with (Fig. 7).

### The Importance of Being Causal

In case of *SERPING1* nonsense and frameshift variants, prematurely introducing a stop codon and/or nonsense mediated decay is generally the assumed pathomechanism. Similarly, there are no pathomechanism doubts in case of whole exon deletions. However, assessing the impact of missense variants and splicing variants located outside canonical splicing positions (± 1,2) is a more demanding process, as only a few functional studies are available (for variants assessed by functional test(s) see Tables 2, 3, 4, and 5). Therefore, clinically based databases like HGMD [74] or LOVD [76] play crucial roles in providing information on reported cases carrying the same variant which is very important when applying ACMG based variant classification. Further, it is noteworthy that the ClinGen Variant Curation Expert Panel has begun to investigate HAE [79].

Even though mRNA analysis might sometimes be strenuous due to the small extracted quantity of *SERPING1* mRNA from the whole blood [19], PCR of cDNA designed to detect a specific splicing defect followed by capillary electrophoresis still presents the first-choice methodology in our hands. Using this procedure, we were able to detect aberrant transcripts in two related patients carrying the c.686-7C > G variant. In this case, the aberrant transcript was not degraded by NMD; however, even in NMD-driven degradation, capillary electrophoresis appears to be sensitive enough to detect the aberrant transcript [19].

Specific splicing in silico prediction tools may help specify the defect and draw attention in the right direction. However, splicing variants’ impact outside canonical GT or AG dinucleotides is sometimes difficult to assess by these tools as, for instance, the MaxEnt Score often does not decrease substantially. Therefore, using a minigene system can provide invaluable information especially if the patient’s RNA is unavailable.

For example, minigene analysis of c.550 + 3A > C confirmed exon 3 splicing disruption. However, it is important to carefully interpret the test results. In this variant, the transcript created by retaining part of intron 3 makes a substantial part of the detected mutant minigene transcripts but a similar transcript would not occur in vivo at all [5]. This difference emerges from simplifying the genomic context in a minigene, where the intron downstream of studied exon is shortened from 1657 to 530 bp only, which makes intron retention more probable compared to real *SERPING1*. Thus, we would primarily expect exon skipping and cryptic 5'ss use in a patient carrying this variant.

With as many as 20% of causal de novo variants, *SERPING1* is regarded as a mutagenic liability, possibly due to its location near the centromeric region and presence of CpG islands in the coding region. Nevertheless, it still might be useful to monitor a particular population even with such a high sequence variation rate. We found few variants that occur specifically in the Czech cohort. The most common variant in our HAE-1 cohort—p.Val454Gly—previously described only in one patient in our other study, was additionally found in three other pedigrees. Similarly, the variant c.1284_1285del, which was previously reported only in one Czech pedigree [18], was discovered in two additional families. Furthermore, another deep intronic variant, c.1029 + 384A > G, was detected in three families, however, this variant’s incidence in other populations might still be underestimated because the variant location is usually not routinely analyzed by Sanger sequencing and targeted or exome NGS [71, 72]. Beside these variants, others were specific to one or two families except for the HAE-2 variants in active center and large deletions.

### Severity of HAE

The HAE phenotype severity ranges from asymptomatic to very severe and even members of the same family carrying the same *SERPING1* alleles have a very distinct disease. Numerous studies have investigated the correlation between causal variant types and phenotype, adopting diverse approaches for variant classification and phenotype characterization. In several studies, variants were categorized into two groups—first comprising nonsense, frameshift, large deletion/insertions, splicing defects and HAE-2 variants, and second missense variants excluding HAE-2 variants [44, 50, 80], and whereas Andrejevic et al. and Grivčeva-Panovska et al. [44, 80] found that the first group of variants correlated with worse clinical severity score, Maia et al. [50] found no correlation with the phenotype. Similar to our approach, Speletas et al. [12] considered HAE-2 variants a specific entity and compared HAE-1 missense variants to null variants, and similarly to our results, they found association between missense variant and later onset of the HAE.

Duponchel et al. [66] showed that the c.-21 T > C variant causes partial exon 2 skipping. It has been suggested that even though it is not causal in heterozygous carriers, it may still potentially cause mild HAE in a homozygous state [48] and, in *trans* position to another causal *SERPING1* variant, may be linked to a more severe clinical manifestation [35, 51, 81]. We detected no homozygous c.-21 T > C carrier in our cohort. However, we did examine its potential influence on HAE severity and, indeed, found a significant association between c.-21 T > C in *trans* position with another causal variant and a higher number of attacks per year, a lower age at disease onset, as well as a higher Clinical severity score [28].

Although our study comprises the largest reported number of patients with c.-21 T > C in *trans* with another causal variant to the best of our knowledge, it would still be useful to collect and analyze data from several databases, preferably in the form of a multicenter international study, to get a clearer picture of the association between this variant and HAE phenotype.

## Conclusion

Most of the HAE genetic causes are determined by routinely used approaches, such as direct *SERPING1* sequencing of exons, exon/intron boundaries, as well as determining CNVs. When no causal variant is identified by these conventional methods, further molecular genetic techniques should be applied in order to discover the pathogenic alteration in the background of the disease. Primarily, we suggest sequencing intronic and UTR parts of the gene, where pathologic variants have been previously reported, then, analyzing mRNA ideally in several affected and unaffected family members, and/or performing functional minigene tests, if a variant of unknown significance is found. Using targeted panel sequencing, which is becoming standard, we can analyze all the *SERPING1* regions, as well as other genes associated with HAE in one step.

As demanding as the procedure of uncovering the possible underlying defect might appear, functional analysis and correct interpretation of the variant pathogenicity often presents an even more substantial challenge. Even though we have provided an experimental insight into the pathomechanism of some splicing variants in previously published studies as well as in this paper, several variants possibly affecting *SERPING1* expression and splicing still await functional evidence.

### Supplementary Information

Below is the link to the electronic supplementary material.

Supplementary file1 (PDF 846 KB)

## Data Availability

The datasets analyzed during the current study are available in the Supplement; more detailed information is available from the corresponding author on reasonable request.
