# Supplementary material for: Systematic Approach Revealed SERPING1 Splicing-Affecting Variants to be Highly Represented in the Czech National HAE Cohort
Source: J Clin Immunol. 2023 Aug 25;43(8):1974–91. doi: 10.1007/s10875-023-01565-w (PMC10661775; doi:10.1007/s10875-023-01565-w)
Supplement: Supplementary file 1 — Supplementary file1 (PDF 846 KB) [file 10875_2023_1565_MOESM1_ESM.pdf]

# Systematic approach revealed *SERPING1* splicing-affecting variants to be highly represented in the Czech national HAE cohort

**Grombirikova H. et al.**

## Supplementary material

- Tables S1-S3
- Supplementary methods
- Figures S1, S2

| Sample ID | Year of birth | Causal variant | Protein change | Method | c.-21T>C | Symptomatic/Asymptomatic | Age of onset | Age of diagnosis | No. of edemas per year | Long term prophylaxis | Clinical severity score | C1-INH [g/l] | C1-INH Normal values [g/l] | C1-INH evaluation | C1-INH function [%] | C1-INH func. Normal values [%] | C1-INH function evaluation | C4 [g/l] | C4 Normal values [g/l] | C4 evaluation |
|---|---|---|---|---|---|---|---|---|---|---|---|---|---|---|---|---|---|---|---|---|
| P00101[#] | 1943 | c.855_856del | p.(Arg286Profs*18) | DGGE + NGS | not | S | 2 | 49 | 24 | | | 0.063 | 0.2-0.35 | L | | | | 0.04 | 0.15-0.4 | L |
| P00102[#] | 1965 | c.855_856del | p.(Arg286Profs*18) | DGGE + Sanger | NA | S | 21 | 27 | 12 | | | 0.064 | 0.2-0.35 | L | | | | 0.07 | 0.2-0.5 | L |
| P00201[#] | 1975 | c.1249+5G>A | | DGGE + Sanger | not | S | 13 | 22 | 24 | | | 0.069 | 0.2-0.35 | L | | | | 0.07 | 0.1-0.4 | L |
| P00301[#] | 1946 | c.685+2_685+13del | | DGGE + Sanger | not | S | 13 | 31 | 30 | yes | 8 | 0.054 | 0.15-0.35 | L | | | | 0.05 | 0.1-0.4 | L |
| P00302 | 1975 | c.685+2_685+13del | | DGGE + Sanger | TRANS | S | 3 | 3 | 25 | yes | 10 | 0.068 | 0.25-0.34 | L | | | | 0.06 | 0.1-0.4 | L |
| P00303 | 1975 | c.685+2_685+13del | | DGGE + Sanger | not | S | 30 | 3 | 1 | yes | 5 | 0.04 | 0.15-0.35 | L | | | | 0.06 | 0.1-0.4 | L |
| P00304 | 2006 | c.685+2_685+13del | | Sanger | not | NA | | | | no | 2 | | | | | | | | | |
| P00401[#] | 1947 | c.1361T>G, c.564_569dup[†] | p.Val454Gly, p.Asn188_Thr189dup[†] | DGGE + Sanger | NA | S | 21 | 40 | 14 | yes | 5 | 0.137 | 0.25-0.34 | L | | | | 0.06 | 0.1-0.34 | L |
| P00501[#] | 1947 | c.1322T>A | p.Met441Lys | DGGE + Sanger | not | S | 6 | 42 | 15 | yes | 9 | 0.05 | 0.15-0.35 | L | 44 | >68 | L | 0.06 | 0.14-0.35 | L |
| P00502 | 1969 | c.1322T>A | p.Met441Lys | DGGE + Sanger | not | S | 19 | 19 | 12 | yes | 7 | 0.07 | 0.15-0.35 | L | 57 | >68 | L | 0.06 | 0.14-0.35 | L |
| P00503 | 2001 | c.1322T>A | p.Met441Lys | DGGE + NGS | not | S | | 6 | 2 | no | 3 | 0.04 | 0.15-0.35 | L | 61 | >68 | L | 0.1 | 0.14-0.35 | L |
| P00601 | 1958 | c.305_317del | p.(Pro102Leufs*42) | DGGE + Sanger | not | S | 16 | 20 | 7 | yes | 6 | 0.07 | 0.15-0.35 | L | 22 | >68 | L | 0.05 | 0.1-0.4 | L |
| P00602 | 1957 | c.305_317del | p.(Pro102Leufs*42) | DGGE + Sanger | TRANS | S | 18 | 22 | 25 | yes | 8 | 0.03 | 0.15-0.35 | L | 15 | >68 | L | 0.02 | 0.1-0.4 | L |
| P00603 | 1977 | c.305_317del | p.(Pro102Leufs*42) | DGGE + Sanger | not | S | 6 | 15 | 13 | yes | 8 | 0.037 | 0.25-0.34 | L | 21 | >68 | L | 0.03 | 0.14-0.35 | L |
| P00604 | 2011 | c.305_317del | p.(Pro102Leufs*42) | Sanger | not | A | | 2 | | no | | 0.05 | 0.15-0.36 | L | | | | 0.04 | 0.14-0.35 | L |
| P00605 | 1934 | c.305_317del | p.(Pro102Leufs*42) | DGGE + Sanger | not | S | 19 | | | | | 0.13 | 0.15-0.35 | L | | | | 0.07 | 0.1-0.4 | L |
| P00606 | 1972 | c.305_317del | p.(Pro102Leufs*42) | Sanger | NA | S | 28 | 28 | 12 | yes | 7 | 0.03 | 0.15-0.35 | L | 0 | >68 | L | 0.02 | 0.1-0.4 | L |
| P00607 | 1998 | c.305_317del | p.(Pro102Leufs*42) | Sanger | not | S | 16 | 16 | 10 | yes | 8 | 0.04 | 0.15-0.35 | L | 22 | >68 | L | 0.03 | 0.1-0.4 | L |
| P00701[#] | 1973 | c.1202T>A | p.Ile401Asn | DGGE + Sanger | not | S | 10 | 10 | 8 | yes | 7 | 0.04 | 0.15-0.35 | L | 18 | >68 | L | 0.04 | 0.14-0.34 | L |
| P00702 | 1951 | c.1202T>A | p.Ile401Asn | DGGE + Sanger | not | S | 10 | 35 | 3 | yes | | 0.05 | 0.15-0.35 | L | 57 | >68 | L | 0.1 | 0.14-0.35 | L |
| P00801[#] | 1974 | c.793T>G | p.Trp265Gly | DGGE + Sanger | not | S | 19 | 23 | 6 | yes | 8 | 0.018 | 0.15-0.35 | L | 52 | >68 | L | 0.02 | 0.15-0.40 | L |
| P00802 | 1948 | c.793T>G | p.Trp265Gly | DGGE + Sanger | not | S | 25 | 58 | 2 | no | 3 | 0.043 | 0.15-0.35 | L | 61 | >68 | L | 0.02 | 0.15-0.40 | L |
| P00803 | 1958 | c.793T>G | p.Trp265Gly | DGGE + Sanger | not | S | 16 | 48 | 5 | no | 7 | 0.038 | 0.15-0.35 | L | 0 | >68 | L | 0.02 | 0.15-0.40 | L |

| Sample ID | Year of birth | Causal variant | Protein change | Method | c.-21T>C | Symptomatic/Asymptomatic | Age of onset | Age of diagnosis | No. of edemas per year | Long term prophylaxis | Clinical severity score | C1-INH [g/l] | C1-INH Normal values [g/l] | C1-INH evaluation | C1-INH function [%] | C1-INH func. Normal values [%] | C1-INH function evaluation | C4 [g/l] | C4 Normal values [g/l] | C4 evaluation |
|---|---|---|---|---|---|---|---|---|---|---|---|---|---|---|---|---|---|---|---|---|
| P00901# | 1966 | c.1115del | p.(Gln372Argfs*25) | DGGE + Sanger | TRANS | S | 4 | 4 | 27 | yes | 10 | 0.08 | 0.15-0.35 | L | 31 | >68 | L | 0.02 | 0.14-0.35 | L |
| P00902 | 1994 | c.1115del | p.(Gln372Argfs*25) | DGGE + Sanger | not | S | 8 | | 23 | yes | 7 | 0.05 | 0.15-0.35 | L | 29 | >68 | L | 0.02 | 0.14-0.35 | L |
| P01001 | 1985 | c.1396C>T | p.Arg466Cys | DGGE + Sanger | not | S | 5 | 5 | 24 | yes | 10 | 0.47 | 0.15-0.35 | H | 49 | >68 | L | 0.1 | 0.14-0.35 | L |
| P01002 | 2008 | c.1396C>T | p.Arg466Cys | Sanger | not | S | 4 | 4 | 6 | no | 6 | 0.43 | 0.15-0.35 | H | 55 | >68 | L | 0.08 | 0.14-0.35 | L |
| P01003 | 2011 | c.1396C>T | p.Arg466Cys | DGGE + Sanger | not | S | 6 | 1 | 6 | no | 5 | 0.42 | 0.15-0.35 | H | 45 | >68 | L | 0.1 | 0.14-0.35 | L |
| P01101# | 1986 | c.1225_1249+19del | | Sanger | not | S | 15 | 17 | 39 | yes | 8 | 0.04 | 0.21-0.39 | L | 24 | >68 | L | 0.019 | 0.1-0.4 | L |
| P01102 | 2004 | c.1225_1249+19del | | Sanger | not | S | 6 | 1 | 2 | no | 4 | 0.076 | 0.21-0.39 | L | 37 | >68 | L | 0.05 | 0.1-0.38 | L |
| P01201# | 1957 | c.1046T>C | p.Leu349Pro | DGGE + Sanger | not | S | 27 | 47 | 14 | yes | 4 | 0.05 | 0.15-0.35 | L | | | | 0.05 | 0.14-0.34 | L |
| P01202 | 2002 | c.1046T>C | p.Leu349Pro | DGGE + Sanger | not | S | 9 | 10 | 6 | no | 5 | 0.05 | 0.15-0.35 | L | 40 | >68 | L | 0.05 | 0.1-0.4 | L |
| P01301# | 2004 | c.550G>A | p.Gly184Arg | Sanger | not | S | 7 | 9 | 20 | yes | 8 | 0.07 | 0.15-0.36 | L | 29 | >68 | L | 0.03 | 0.14-0.35 | L |
| P01401 | 1984 | c.1284_1285del | p.(Cys428Trpfs*44) | Sanger | not | S | 17 | 21 | 27 | yes | 8 | 0.095 | 0.15-0.35 | L | 48 | >68 | L | 0.11 | 0.15-0.40 | L |
| P01501 | 1975 | c.743C>G | p.Pro248Arg | Sanger | not | S | 25 | 35 | 5 | yes | 6 | 0.1 | 0.15-0.36 | L | 39 | >68 | L | 0.05 | 0.14-0.35 | L |
| P01502 | 2006 | c.743C>G | p.Pro248Arg | Sanger | not | S | 6 | 10 | 3 | no | | 0.09 | 0.15-0.36 | L | 57 | >68 | L | 0.03 | 0.14-0.35 | L |
| P01503 | 2009 | c.743C>G | p.Pro248Arg | Sanger | not | S | 8 | 7 | 4 | no | 4 | 0.08 | 0.15-0.36 | L | 44 | >68 | L | 0.05 | 0.14-0.35 | L |
| P01601 | 1958 | EX7del | | MLPA | not | NA | | | | | | | | | | | | | | |
| P01602 | 1981 | EX7del | | MLPA | not | S | | | 9 | no | 2 | 0.031 | 0.21-0.33 | L | 15 | >68 | L | 0.02 | 0.1-0.38 | L |
| P01603 | 2013 | EX7del | | MLPA | not | A | | 6 | | | | 0.03 | 0.21-0.34 | L | 21 | >68 | L | 0.02 | 0.1-0.38 | L |
| P01604 | 1986 | EX7del | | MLPA | not | NA | | | | no | 5 | | | | | | | | | |
| P01701 | 1981 | variant not detected | | | ND | S | 23 | 35 | 2 | no | | 0.059 | 0.21-0.39 | L | 36 | >68 | L | 0.08 | 0.1-0.38 | L |
| P01901 | 1992 | c.506T>C | p.Phe169Ser | Sanger | not | S | 18 | 18 | 1 | no | | 0.07 | 0.15-0.36 | L | 30 | >68 | L | 0.04 | 0.14-0.35 | L |
| P01902 | 2021 | c.506T>C | p.Phe169Ser | Sanger | not | NA | | | | | | | | | | | | | | |
| P02001 | 1985 | c.1397G>A | p.Arg466His | Sanger | not | S | | 32 | 6 | no | | 0.36 | 0.15-0.36 | N | 46 | >68 | L | 0.13 | 0.14-0.35 | L |
| P02101 | 1994 | EX1-8del | | MLPA | not | S | 14 | 23 | 9 | no | 6 | 0.047 | 0.21-0.39 | L | 20 | >68 | L | 0.05 | 0.1-0.38 | L |
| P02201 | 1994 | c.550+3A>C | | Sanger | not | S | 16 | 23 | 24 | yes | 6 | 0.05 | 0.15-0.35 | L | 38 | >68 | L | 0.08 | 0.1-0.4 | L |
| P02301 | 1965 | c.51+5G>A | | Sanger | not | S | 14 | 24 | 5 | no | 2 | 0.086 | 0.21-0.33 | L | 27 | >68 | L | 0.1 | 0.1-0.38 | N |
| P02401 | 1973 | EX4del | | MLPA | not | NA | | | | | | | | | | | | | | |
| P02501 | 1961 | c.1396C>T | p.Arg466Cys | Sanger | not | S | 20 | 58 | 15 | yes | 4 | 0.284 | 0.15-0.35 | N | 37 | >68 | L | | | |
| P02502 | 1987 | c.1396C>T | p.Arg466Cys | Sanger | not | S | 33 | 34 | 1 | no | | 0.45 | 0.15-0.35 | H | 67 | >68 | L | 0.14 | 0.15-0.40 | L |
| P02503 | 1991 | c.1396C>T | p.Arg466Cys | Sanger | not | S | 15 | 29 | 3 | no | 3 | 0.439 | 0.15-0.35 | H | 51 | >68 | L | | | |
| P02601 | 1962 | c.706T>G | p.Phe236Val | DGGE + Sanger | not | NA | | | | | | | | | | | | | | |
| P02602# | 1994 | c.706T>G | p.Phe236Val | Sanger | NA | S | 6 | 12 | 25 | yes | 9 | 0.06 | 0.15-0.36 | L | 63 | >68 | L | 0.05 | 0.14-0.35 | L |
| P02701 | 1979 | c.1397G>A | p.Arg466His | Sanger | not | S | 25 | 40 | 5 | no | 2 | 0.357 | 0.21-0.39 | N | 37 | >68 | L | 0.06 | 0.1-0.38 | L |
| P02702 | 2011 | c.1397G>A | p.Arg466His | Sanger | not | A | | 8 | | | | 0.35 | 0.21-0.39 | N | 75 | >68 | N | 0.06 | 0.1-0.38 | L |
| P02801 | 2011 | c.686-1G>T | | Sanger | ND | S | 4 | 8 | 17 | no | 9 | 0.047 | 0.21-0.39 | L | 31 | >68 | L | 0.05 | 0.1-0.38 | L |
| P02901 | 1977 | c.1029+384A>G | | Sanger | TRANS | S | 21 | 42 | 2 | no | 1 | 0.064 | 0.15-0.35 | L | 32 | >68 | L | 0.08 | 0.14.0.35 | L |
| P02902 | 1949 | c.1029+384A>G | | NGS | not | S | 72 | 72 | 3 | | | 0.07 | 0.21-0.39 | L | 41 | >68 | L | 0.11 | 0.1-0.38 | N |
| P03001 | 1976 | c.686-7C>G | | Sanger | not | S | 15 | 43 | 1 | no | 3 | 0.058 | 0.21-0.39 | L | 31 | >68 | L | 0.05 | 0.1-0.38 | L |
| P03002 | 2002 | c.686-7C>G | | Sanger | not | S | 10 | 18 | 4 | no | 5 | 0.05 | 0.21-0.39 | L | 25 | >68 | L | 0.03 | 0.1-0.38 | L |
| P03101 | 1984 | c.722G>C | p.Arg241Pro | Sanger | not | S | 34 | 35 | 2 | no | 3 | 0.115 | 0.15-0.35 | L | 43 | >68 | L | 0.07 | 0.15-0.40 | L |
| P03102 | 1945 | c.722G>C | p.Arg241Pro | Sanger | not | S | 32 | 75 | 1 | no | 1 | 0.121 | 0.15-0.35 | L | 58 | >68 | L | 0.1 | 0.15-0.41 | L |
| P03301 | 1971 | EX4del | | MLPA | not | S | 16 | 49 | 16 | no | 5 | 0.044 | 0.21-0.39 | L | 32 | >68 | L | 0.05 | 0.1-0.38 | L |
| P03401# | 1970 | c.600dup | p.(Lys201Glnfs*56) | DGGE + Sanger | not | S | 14 | | 24 | yes | 5 | | | | | | | | | |
| P03402 | 1942 | c.600dup | p.(Lys201Glnfs*56) | Sanger | not | NA | | | | yes | 4 | | | | | | | | | |
| P03501 | 1987 | c.151_152del | p.(Ser51Glnfs*6) | Sanger | not | S | 5 | 33 | 3 | no | 5 | 0.05 | 0.15-0.36 | L | 31 | >68 | L | 0.02 | 0.14-0.35 | L |
| P03502 | 2019 | c.151_152del | p.(Ser51Glnfs*6) | Sanger | not | S | 2 | 1 | 2 | | | 0.11 | 0.15-0.36 | L | 40 | >68 | L | 0.04 | 0.14-0.35 | L |
| P03701# | 1990 | c.685+1del | | DGGE + Sanger | not | S | 7 | 14 | 20 | yes | 6 | 0.037 | 0.15-0.35 | L | 37 | >68 | L | 0.03 | 0.14-0.35 | L |
| P03702 | 2001 | c.685+1del | | Sanger | not | NA | | | | no | | | | | | | | | | |
| P03703 | 1967 | c.685+1del | | Sanger | not | NA | | | | no | | | | | | | | | | |
| P03704 | 1999 | c.685+1del | | Sanger | not | NA | | | | no | 3 | | | | | | | | | |
| P03801# | 1973 | c.551-2A>G | | DGGE + Sanger | not | S | 10 | 18 | 24 | yes | 5 | 0.054 | 0.21-0.39 | L | 5 | >68 | L | 0.035 | 0.1-0.38 | L |
| P03802 | 1979 | c.551-2A>G | | Sanger | not | S | 17 | 28 | 3 | no | 7 | 0.039 | 0.21-0.39 | L | 38 | >68 | L | 0.023 | 0.1-0.38 | L |
| P03803 | 2013 | c.551-2A>G | | Sanger | not | S | 6 | 2 | 3 | no | | 0.04 | 0.21-0.39 | L | 20 | >68 | L | 0.02 | 0.1-0.38 | L |
| P03804 | 2000 | c.551-2A>G | | Sanger | not | S | 9 | 11 | 9 | yes | 8 | 0.053 | 0.21-0.39 | L | 39 | >68 | L | 0.054 | 0.1-0.38 | L |

| Sample ID | Year of birth | Causal variant | Protein change | Method | c.-21T>C | Symptomatic/Asymptomatic | Age of onset | Age of diagnosis | No. of edemas per year | Long term prophylaxis | Clinical severity score | C1-INH [g/l] | C1-INH Normal values [g/l] | C1-INH evaluation | C1-INH function [%] | C1-INH func. Normal values [%] | C1-INH function evaluation | C4 [g/l] | C4 Normal values [g/l] | C4 evaluation |
|---|---|---|---|---|---|---|---|---|---|---|---|---|---|---|---|---|---|---|---|---|
| P03805 | 1998 | c.551-2A>G | | Sanger | not | S | 11 | 13 | 15 | yes | 5 | 0.034 | 0.21-0.39 | L | 27 | >68 | L | 0.03 | 0.1-0.38 | L |
| P03806 | 1948 | c.551-2A>G | | Sanger | not | S | 30 | 59 | 6 | | | 0.066 | 0.21-0.39 | L | 57 | >68 | L | 0.101 | 0.1-0.38 | N |
| P03807 | 2007 | c.551-2A>G | | Sanger | not | S | 7 | 0 | 2 | yes | 4 | 0.077 | 0.21-0.39 | L | 44 | >68 | L | 0.07 | 0.1-0.38 | L |
| P03808 | 2016 | c.551-2A>G | | Sanger | not | S | | 2 | 2 | | | 0.039 | 0.21-0.39 | L | 23 | >68 | L | 0.03 | 0.1-0.38 | L |
| P03901 | 2009 | EX4del | | MLPA | not | S | 6 | 12 | | | | 0.08 | 0.15-0.36 | L | 35 | >68 | L | 0.04 | 0.14-0.35 | L |
| P04001 | 1993 | EX4del | | MLPA | not | S | 12 | 13 | 3 | no | 4 | 0.04 | 0.15-0.36 | L | 11 | >68 | L | 0.03 | 0.14-0.35 | L |
| P04002 | 1964 | EX4del | | MLPA | not | S | 40 | 46 | 4 | no | 4 | 0.06 | 0.15-0.36 | L | 47 | >68 | L | 0.03 | 0.14-0.35 | L |
| P04101 | 1983 | EX4del | | MLPA | not | NA | | | | | | | | | | | | | | |
| P04201[#] | 1984 | c.305_317del | p.(Pro102Leufs*42) | DGGE + Sanger | not | S | 10 | 10 | 10 | yes | 8 | 0.05 | 0.15-0.36 | L | 33 | >68 | L | 0.03 | 0.14-0.35 | L |
| P04202 | 1991 | c.305_317del | p.(Pro102Leufs*42) | Sanger | not | S | 10 | 10 | 13 | yes | 6 | 0.04 | 0.15-0.36 | L | 32 | >68 | L | 0.02 | 0.14-0.35 | L |
| P04203 | 1969 | c.305_317del | p.(Pro102Leufs*42) | Sanger | not | NA | | | | no | 3 | | | | | | | | | |
| P04204 | 2004 | c.305_317del | p.(Pro102Leufs*42) | Sanger | not | S | 9 | 12 | 2 | no | | 0.07 | 0.15-0.36 | L | 30 | >68 | L | 0.05 | 0.14-0.35 | L |
| P04205 | 1963 | c.305_317del | p.(Pro102Leufs*42) | Sanger | not | S | 17 | 39 | 2 | no | 3 | 0.03 | 0.15-0.35 | L | 31 | >68 | L | 0.03 | 0.14-0.34 | L |
| P04206 | 1990 | c.305_317del | p.(Pro102Leufs*42) | Sanger | not | S | 13 | 13 | 28 | no | 4 | 0.04 | 0.15-0.35 | L | 22 | >68 | L | 0.02 | 0.14-0.34 | L |
| P04207 | 2016 | c.305_317del | p.(Pro102Leufs*42) | Sanger | not | A | | | | no | | 0.12 | 0.15-0.35 | L | 56 | >68 | L | 0.08 | 0.14-0.34 | L |
| P04301[#] | 1978 | c.686-12A>G | | DGGE + Sanger | not | S | 10 | 30 | 7 | yes | 5 | 0.057 | 0.21-0.39 | L | 44 | >68 | L | 0.066 | 0.1-0.38 | L |
| P04401 | 1997 | EX4del | | MLPA | not | NA | | | | | | | | | | | | | | |
| P04402 | 1967 | EX4del | | MLPA | not | NA | | | | | | | | | | | | | | |
| P04501 | 1977 | c.-22-19_-22-4del | | DGGE + Sanger | not | S | 23 | | 12 | yes | 4 | 0.06 | 0.15-0.36 | L | 44 | >68 | L | 0.03 | 0.14-0.35 | L |
| P04502 | 2000 | c.-22-19_-22-4del | | Sanger | not | A | | 13 | | no | | 0.06 | 0.15-0.36 | L | 62 | >68 | L | 0.05 | 0.14-0.35 | L |
| P04601[#] | 1994 | c.1283del | p.(Cys428Leufs*3) | DGGE + Sanger | not | S | 4 | 15 | 11 | yes | 8 | 0.089 | 0.15-0.35 | L | 43 | >68 | L | 0.068 | 0.1-0.38 | L |
| P04701 | 1967 | EX7del | | MLPA | not | S | 15 | 41 | 6 | yes | 6 | 0.048 | 0.15-0.35 | L | 49 | >68 | L | 0.06 | 0.15-0.40 | L |
| P04702 | 2002 | EX7del | | MLPA | not | S | 17 | 6 | 2 | no | 4 | 0.095 | 0.15-0.35 | L | 38 | >68 | L | 0.06 | 0.15-0.40 | L |
| P04801[#] | 1979 | c.897G>A | p.(Trp299*) | DGGE + Sanger | TRANS | S | 3 | 10 | 17 | yes | 8 | 0.03 | 0.15-0.35 | L | 37 | >68 | L | 0.02 | 0.1-0.4 | L |
| P04802 | 1957 | c.897G>A | p.(Trp299*) | Sanger | not | S | | 30 | 5 | yes | 2 | 0.05 | 0.15-0.36 | L | 36 | >68 | L | 0.09 | 0.14-0.35 | L |
| P04803 | 2010 | c.897G>A | p.(Trp299*) | Sanger | not | S | 4 | 1 | 14 | no | 6 | 0.09 | 0.15-0.35 | L | 36 | >68 | L | 0.07 | 0.14-0.34 | L |
| P04901 | 1968 | c.551-2A>G | | DGGE + Sanger | not | S | 10 | 28 | 15 | yes | 9 | 0.06 | 0.21-0.39 | L | 60 | >68 | L | 0.03 | 0.1-0.38 | L |
| P04902 | 1991 | c.551-2A>G | | DGGE + Sanger | not | S | 17 | 17 | 17 | yes | 8 | 0.066 | 0.21-0.39 | L | 40 | >68 | L | 0.089 | 0.1-0.38 | L |
| P04903 | 2006 | c.551-2A>G | | Sanger | not | S | 8 | 8 | 4 | no | 3 | 0.038 | 0.21-0.39 | L | 32 | >68 | L | 0.03 | 0.1-0.38 | L |
| P05001 | 1993 | c.1397G>A | p.Arg466His | DGGE + Sanger | TRANS | S | 13 | 16 | 60 | yes | 8 | 0.333 | 0.15-0.35 | N | 56 | >68 | L | 0.055 | 0.15-0.40 | L |
| P05002 | 2016 | c.1397G>A | p.Arg466His | Sanger | TRANS | S | 2 | 2 | 4 | no | 6 | 0.383 | 0.15-0.35 | H | 27 | >68 | L | 0.03 | 0.15-0.40 | L |
| P05003 | 1970 | c.1397G>A | p.Arg466His | DGGE + Sanger | not | S | 23 | 39 | 7 | yes | 7 | 0.369 | 0.15-0.35 | H | 27 | >68 | L | 0.07 | 0.15-0.40 | L |
| P05004 | 1988 | c.1397G>A | p.Arg466His | Sanger | not | S | 30 | 26 | 1 | no | 1 | 0.373 | 0.15-0.35 | H | 42 | >68 | L | 0.05 | 0.15-0.40 | L |
| P05101 | 1965 | c.1361T>G | p.Val454Gly | DGGE + Sanger | not | S | 22 | 44 | 4 | yes | 4 | 0.158 | 0.21-0.39 | L | 44 | >68 | L | 0.053 | 0.1-0.38 | L |
| P05102 | 1994 | c.1361T>G | p.Val454Gly | Sanger | not | A | | 19 | | no | | 0.164 | 0.21-0.39 | L | 25 | >68 | L | 0.06 | 0.1-0.38 | L |
| P05103 | 1961 | c.1361T>G | p.Val454Gly | DGGE + Sanger | not | S | 17 | 36 | 3 | no | 7 | 0.176 | 0.21-0.39 | L | 18 | >68 | L | 0.088 | 0.1-0.38 | L |
| P05104 | 1993 | c.1361T>G | p.Val454Gly | Sanger | not | S | 18 | 18 | 1 | yes | 3 | 0.2 | 0.21-0.39 | L | 54 | >68 | L | 0.092 | 0.1-0.38 | L |
| P05201 | 1973 | c.1460_1466del | p.(Lys487Metfs*87) | DGGE + Sanger | not | S | 19 | 36 | 32 | yes | 8 | 0.03 | 0.15-0.35 | L | 0 | >68 | L | 0.02 | 0.14-0.34 | L |
| P05202 | 1949 | c.1460_1466del | p.(Lys487Metfs*87) | Sanger | not | S | 14 | 60 | 24 | yes | 8 | 0.03 | 0.15-0.35 | L | 0 | >68 | L | 0.02 | 0.1-0.4 | L |
| P05203 | 2010 | c.1460_1466del | p.(Lys487Metfs*87) | Sanger | not | S | 4 | 2 | 20 | no | 9 | 0.06 | 0.15-0.35 | L | 15 | >68 | L | 0.06 | 0.14-0.34 | L |
| P05204 | 1972 | c.1460_1466del | p.(Lys487Metfs*87) | Sanger | not | S | 13 | 37 | 20 | yes | 6 | 0.05 | 0.15-0.35 | L | 0 | >68 | L | 0.04 | 0.1-0.4 | L |
| P05205 | 2010 | c.1460_1466del | p.(Lys487Metfs*87) | Sanger | not | S | 2 | 1 | 4 | no | 9 | 0.04 | 0.15-0.35 | L | 46 | >68 | L | 0.06 | 0.1-0.4 | L |
| P05301 | 1976 | c.795_796delGGinsT, c.785T>C[†] | p.(Trp265Cysfs*14), p.Ile262Thr[†] | DGGE + Sanger | not | S | 15 | 33 | 25 | yes | 6 | 0.084 | 0.21-0.39 | L | 61 | >68 | L | 0.038 | 0.1-0.38 | L |
| P05302 | 2002 | c.795_796delGGinsT, c.785T>C[†] | p.(Trp265Cysfs*14), p.Ile262Thr[†] | DGGE + Sanger | not | S | 9 | 8 | 1 | no | 4 | 0.041 | 0.21-0.39 | L | 20 | >68 | L | 0.05 | 0.1-0.38 | L |
| P05401 | 1992 | c.1249+1G>A | | DGGE + Sanger | not | S | 13 | 17 | 12 | yes | 4 | 0.13 | 0.15-0.36 | L | 53 | >68 | L | 0.07 | 0.14-0.35 | L |
| P05501 | 2001 | c.726_777del | p.(Leu243Serfs*19) | DGGE + Sanger | not | S | 9 | 10 | 6 | no | 5 | 0.038 | 0.15-0.35 | L | 32 | >68 | L | 0.02 | 0.15-0.40 | L |

| Sample ID | Year of birth | Causal variant | Protein change | Method | c.-21T>C | Symptomatic/Asymptomatic | Age of onset | Age of diagnosis | No. of edemas per year | Long term prophylaxis | Clinical severity score | C1-INH [g/l] | C1-INH Normal values [g/l] | C1-INH evaluation | C1-INH function [%] | C1-INH func. Normal values [%] | C1-INH function evaluation | C4 [g/l] | C4 Normal values [g/l] | C4 evaluation |
|---|---|---|---|---|---|---|---|---|---|---|---|---|---|---|---|---|---|---|---|---|
| P05502 | 1992 | c.726_777del | p.(Leu243Serfs*19) | Sanger | not | S | 20 | 25 | 4 | yes | 5 | 0.083 | 0.15-0.35 | L | 59 | >68 | L | 0.06 | 0.15-0.40 | L |
| P05503 | 1970 | c.726_777del | p.(Leu243Serfs*19) | Sanger | not | S | 7 | 41 | 1 | yes | 6 | 0.083 | 0.15-0.35 | L | 54 | >68 | L | 0.08 | 0.15-0.40 | L |
| P05504 | 1975 | c.726_777del | p.(Leu243Serfs*19) | Sanger | not | S | 15 | 36 | 4 | no | 4 | 0.128 | 0.15-0.35 | L |  |  |  | 0.12 | 0.15-0.40 | L |
| P05505 | 1941 | c.726_777del | p.(Leu243Serfs*19) | Sanger | not | S | 68 | 73 | 1 | no | 2 | 0.181 | 0.15-0.35 | N | 71 | >68 | N | 0.23 | 0.15-0.40 | N |
| P05506 | 1996 | c.726_777del | p.(Leu243Serfs*19) | Sanger | not | S | 22 |  | 1 | no |  | 0.063 | 0.21-0.39 | L | 48 | >68 | L | 0.06 | 0.1-0.38 | L |
| P05507 | 2000 | c.726_777del | p.(Leu243Serfs*19) | Sanger | not | S | 8 | 9 | 22 | yes | 7 | 0.054 | 0.15-0.35 | L | 26 | >68 | L | 0.04 | 0.15-0.40 | L |
| P05601 | 1975 | c.1361T>G | p.Val454Gly | DGGE + Sanger | not | S | 19 | 36 | 33 | yes | 8 | 0.153 | 0.21-0.39 | L | 41 | >68 | L | 0.09 | 0.1-0.38 | L |
| P05602 | 2002 | c.1361T>G | p.Val454Gly | Sanger | not | A |  | 10 |  | no |  | 0.168 | 0.21-0.39 | L | 54 | >68 | L | 0.092 | 0.1-0.38 | L |
| P05701 | 1984 | c.503C>A | p.Ala168Asp | DGGE + Sanger | CIS | S | 26 | 27 | 55 | no | 6 | 0.042 | 0.21-0.39 | L | 24 | >68 | L | 0.034 | 0.1-0.38 | L |
| P05702 | 2016 | c.503C>A | p.Ala168Asp | Sanger | CIS | A |  | 3 |  |  |  | 0.085 | 0.21-0.39 | L | 57 | >68 | L | 0.04 | 0.1-0.38 | L |
| P05703 | 2019 | c.503C>A | p.Ala168Asp | Sanger | CIS | A |  | 2 |  |  |  | 0.144 | 0.21-0.39 | L | 70 | >68 | N | 0.11 | 0.1-0.38 | N |
| P05801 | 1976 | c.1249+2T>C |  | DGGE + Sanger | not | S | 18 | 36 | 2 | yes | 3 | 0.04 | 0.15-0.36 | L | 40 | >68 | L | 0.06 | 0.14-0.35 | L |
| P05901 | 1976 | c.1346T>C | p.Leu449Pro | DGGE + Sanger | NA | S | 6 |  | 21 | yes | 9 | 0.05 | 0.15-0.36 | L | 35 | >68 | L | 0.05 | 0.14-0.35 | L |
| P06001 | 1965 | EX4del |  | MLPA | not | S | 6 | 25 | 26 | yes | 9 | 0.1 | 0.15-0.36 | L | 54 | >68 | L | 0.05 | 0.14-0.35 | L |
| P06002 | 2006 | EX4del |  | MLPA | not | S | 1 | 6 | 19 | yes | 7 | 0.12 | 0.15-0.36 | L | 44 | >68 | L | 0.11 | 0.14-0.35 | L |
| P06003 | 1988 | EX4del |  | MLPA | not | S | 7 | 5 | 40 | yes | 9 | 0.07 | 0.15-0.36 | L | 60 | >68 | L | 0.07 | 0.14-0.35 | L |
| P06004 | 2018 | EX4del |  | MLPA | not | A |  | 2 |  |  |  | 0.11 | 0.15-0.36 | L | 75 | >68 | N | 0.09 | 0.14-0.35 | L |
| P06101 | 1975 | c.1396C>T | p.Arg466Cys | DGGE + Sanger | not | S | 10 | 37 | 8 | yes | 9 | 0.765 | 0.21-0.39 | H | 29 | >68 | L | 0.042 | 0.1-0.38 | L |
| P06102 | 1952 | c.1396C>T | p.Arg466Cys | DGGE + Sanger | not | S | 23 | 60 | 13 | no | 2 | 0.286 | 0.21-0.39 | N | 26 | >68 | L | 0.06 | 0.1-0.38 | L |
| P06201 | 1976 | EX4del |  | MLPA | not | S | 25 | 25 | 17 | yes | 6 | 0.11 | 0.15-0.36 | L | 48 | >68 | L | 0.03 | 0.14-0.35 | L |
| P06301 | 1977 | c.498C>A | p.Asn166Lys | DGGE + Sanger | TRANS | S | 12 |  | 23 | yes | 8 | 0.05 | 0.15-0.36 | L | 12 | >68 | L | 0.05 | 0.14-0.35 | L |
| P06401 | 1984 | c.1284_1285del | p.(Cys428Trpfs*44) | DGGE + Sanger | not | S |  |  | 1 | yes | 7 | 0.08 | 0.15-0.36 | L | 55 | >68 | L | 0.05 | 0.14-0.35 | L |
| P06501 | 1992 | c.1420C>T | p.(Gln474*) | DGGE + Sanger | not | S | 3 | 19 | 37 | yes | 10 | 0.04 | 0.15-0.36 | L | 28 | >68 | L | 0.07 | 0.14-0.35 | L |
| P06502 | 1965 | c.1420C>T | p.(Gln474*) | Sanger | not | S | 3 | 46 | 6 | yes | 9 | 0.04 | 0.15-0.36 | L | 40 | >68 | L | 0.07 | 0.14-0.35 | L |
| P06503 | 2018 | c.1420C>T | p.(Gln474*) | Sanger | not | S | 3 | 19 | 37 | no |  | 0.04 | 0.15-0.36 | L | 28 | >68 | L | 0.03 | 0.14-0.35 | L |
| P06601 | 1966 | c.1195C>T | p.Pro399Ser | DGGE + Sanger | TRANS | S | 13 | 46 | 30 | yes | 4 | 0.04 | 0.21-0.39 | L | 11 | >68 | L | 0.034 | 0.1-0.38 | L |
| P06602 | 1988 | c.1195C>T | p.Pro399Ser | Sanger | not | A |  | 25 |  |  |  | 0.043 | 0.21-0.39 | L | 20 | >68 | L | 0.1 | 0.1-0.38 | N |
| P06701 | 1972 | c.1361T>G | p.Val454Gly | DGGE + Sanger | not | S |  | 37 | 6 | yes | 4 | 0.21 | 0.15-0.36 | N | 53 | >68 | L | 0.09 | 0.14-0.35 | L |
| P06801 | 1991 | c.1029+384A>G |  | Sanger | not | S | 10 | 10 | 27 | no | 8 | 0.058 | 0.21-0.39 | L | 0 | >68 | L | 0.02 | 0.1-0.38 | L |
| P06901 | 1978 | c.1397G>A | p.Arg466His | Sanger | not | S | 31 | 34 | 36 |  |  | 0.3 | 0.15-0.36 | N | 79 | >68 | N | 0.06 | 0.14-0.35 | L |
| P06902 | 2019 | c.1397G>A | p.Arg466His | Sanger | NA | NA |  |  |  |  |  |  |  |  |  |  |  |  |  |  |
| P06903 | 2017 | c.1397G>A | p.Arg466His | Sanger | NA | NA |  |  |  |  |  |  |  |  |  |  |  |  |  |  |
| P07001 | 1993 | c.614G>A | p.Cys205Tyr | Sanger | not | S | 10 | 17 | 14 | no | 6 | 0.069 | 0.21-0.39 | L | 31 | >68 | L | 0.05 | 0.1-0.38 | L |
| P07002 | 1971 | c.614G>A | p.Cys205Tyr | Sanger | not | S | 43 | 43 | 1 |  |  | 0.075 | 0.21-0.39 | L | 50 | >68 | L | 0.018 | 0.1-0.38 | L |
| P07003 | 1964 | c.614G>A | p.Cys205Tyr | Sanger | not | S | 29 | 49 | 4 | yes | 5 | 0.069 | 0.21-0.39 | L | 33 | >68 | L | 0.07 | 0.1-0.38 | L |
| P07101 | 1992 | c.1036C>T | p.(Gln346*) | Sanger | not | S | 11 | 18 | 22 | yes | 8 | 0.032 | 0.21-0.39 | L | 27 | >68 | L | 0.04 | 0.1-0.38 | L |
| P07102 | 1968 | c.1036C>T | p.(Gln346*) | Sanger | not | S | 23 | 42 | 24 | yes | 7 | 0.037 | 0.21-0.39 | L | 37 | >68 | L | 0.03 | 0.1-0.38 | L |
| P07103 | 1994 | c.1036C>T | p.(Gln346*) | Sanger | not | S | 16 | 11 | 21 | no | 5 | 0.044 | 0.21-0.39 | L | 35 | >68 | L | 0.059 | 0.1-0.38 | L |
| P07201 | 1970 | EX4del |  | MLPA | TRANS | S | 15 | 30 | 7 | no | 6 | 0.07 | 0.21-0.39 | L | 45 | >68 | L | 0.05 | 0.1-0.38 | L |
| P07202 | 1991 | EX4del |  | MLPA | not | S | 13 | 19 | 8 | yes | 8 | 0.068 | 0.21-0.39 | L | 37 | >68 | L | 0.056 | 0.1-0.38 | L |
| P07301[#] | 1969 | c.550G>T | p.Gly184Trp | DGGE + Sanger | not | S | 22 | 29 | 1 | yes | 4 | 0.085 | 0.21-0.39 | L | 43 | >68 | L | 0.1 | 0.1-0.38 | N |
| P07401[#] | 1955 | c.209C>G | p.(Ser70*) | DGGE + RA | not | S | 12 | 26 | 9 | no | 7 | 0.063 | 0.21-0.39 | L | 56 | >68 | L | 0.05 | 0.1-0.38 | L |
| P07501[#] | 1943 | c.1029+384A>G |  | DGGE + Sanger | not | S | 10 | 37 | 24 | no |  | 0.07 | 0.21-0.39 | L | 40 | >68 | L | 0.11 | 0.1-0.38 | N |
| P07502[#] | 2011 | c.1029+384A>G |  | Sanger | not | A |  | 8 |  |  |  | 0.056 | 0.21-0.39 | L | 49 | >68 | L | 0.07 | 0.1-0.38 | L |
| P07503[#] | 1997 | c.1029+384A>G |  | NGS | not | S | 25 | 4 | 1 | no |  | 0.061 | 0.21-0.39 | L | 41 | >68 | L | 0.06 | 0.1-0.38 | L |
| P07504[#] | 1982 | c.1029+384A>G |  | DGGE + Sanger | not | S | 17 | 18 | 7 | no | 7 | 0.032 | 0.21-0.39 | L | 40 | >68 | L | 0.04 | 0.1-0.38 | L |
| P07505[#] | 1981 | c.1029+384A>G |  | DGGE + Sanger | not | S | 12 | 21 | 24 | yes | 8 | 0.049 | 0.21-0.39 | L | 52 | >68 | L | 0.04 | 0.1-0.38 | L |
| P07506[#] | 1967 | c.1029+384A>G |  | Sanger | not | S | 20 | 17 | 4 | yes | 3 | 0.076 | 0.21-0.39 | L | 60 | >68 | L | 0.08 | 0.1-0.38 | L |

| Sample ID | Year of birth | Causal variant | Protein change | Method | c.-21T>C | Symptomatic/Asymptomatic | Age of onset | Age of diagnosis | No. of edemas per year | Long term prophylaxis | Clinical severity score | C1-INH [g/l] | C1-INH Normal values [g/l] | C1-INH evaluation | C1-INH function [%] | C1-INH func. Normal values [%] | C1-INH function evaluation | C4 [g/l] | C4 Normal values [g/l] | C4 evaluation |
|---|---|---|---|---|---|---|---|---|---|---|---|---|---|---|---|---|---|---|---|---|
| P07507# | 1967 | c.1029+384A>G | | DGGE + Sanger | not | S | 16 | 32 | 2 | no | 2 | 0.075 | 0.21-0.39 | L | 62 | >68 | L | 0.07 | 0.1-0.38 | L |
| P07508# | 1975 | c.1029+384A>G | | DGGE + Sanger | not | S | 17 | 24 | 2 | no | 5 | 0.071 | 0.21-0.39 | L | 78 | >68 | N | 0.06 | 0.1-0.38 | L |
| P07509# | 2012 | c.1029+384A>G | | Sanger | not | A | | 5 | | | | 0.092 | 0.21-0.39 | L | 82 | >68 | N | 0.11 | 0.1-0.38 | N |
| P07510# | 1946 | c.1029+384A>G | | DGGE + Sanger | not | S | 21 | 34 | 12 | no | 6 | 0.034 | 0.21-0.39 | L | 31 | >68 | L | 0.02 | 0.1-0.38 | L |
| P07511# | 1993 | c.1029+384A>G | | DGGE + Sanger | not | A | | 8 | | no | | 0.114 | 0.21-0.39 | L | 57 | >68 | L | 0.09 | 0.1-0.38 | L |
| P07512# | 1935 | c.1029+384A>G | | DGGE + Sanger | not | S | 20 | 21 | | yes | | 0.088 | 0.21-0.39 | L | 47 | >68 | L | 0.05 | 0.1-0.38 | L |
| P07601# | 1948 | c.1396C>T | p.Arg466Cys | DGGE + RA | not | S | 18 | 37 | 53 | yes | 8 | 0.705 | 0.21-0.39 | H | 49 | >68 | L | 0.15 | 0.1-0.38 | N |
| P07602# | 1970 | c.1396C>T | p.Arg466Cys | DGGE + RA | not | S | 5 | 26 | 73 | yes | 10 | 0.541 | 0.21-0.39 | H | 45 | >68 | L | 0.03 | 0.1-0.38 | L |
| P07603# | 1971 | c.1396C>T | p.Arg466Cys | DGGE + RA | not | S | 6 | 18 | 23 | yes | 9 | 0.654 | 0.21-0.39 | H | 51 | >68 | L | 0.04 | 0.1-0.38 | L |
| P07701# | 1966 | c.629T>C | p.Leu210Pro | DGGE + Sanger | not | S | 21 | 31 | 1 | no | 5 | 0.069 | 0.21-0.39 | L | 72 | >68 | N | 0.06 | 0.1-0.38 | N |
| P07702 | 1986 | c.629T>C | p.Leu210Pro | DGGE + RA | not | S | 20 | 20 | 1 | no | 3 | 0.085 | 0.21-0.39 | L | 73 | >68 | N | 0.046 | 0.1-0.38 | L |
| P07801# | 1966 | c.1396C>T | p.Arg466Cys | DGGE + Sanger | not | S | 19 | 26 | 9 | no | 7 | 0.679 | 0.21-0.39 | H | 48 | >68 | L | 0.09 | 0.1-0.38 | L |
| P07802 | 1988 | c.1396C>T | p.Arg466Cys | Sanger | not | S | 5 | 8 | 40 | yes | 10 | 0.643 | 0.21-0.39 | H | 42 | >68 | L | 0.019 | 0.1-0.38 | L |
| P07803# | 1946 | c.1396C>T | p.Arg466Cys | DGGE + Sanger | not | S | 3 | 45 | 14 | yes | 9 | 0.694 | 0.21-0.39 | H | 65 | >68 | L | 0.14 | 0.1-0.38 | N |
| P07804 | 2017 | c.1396C>T | p.Arg466Cys | Sanger | not | S | 5 | 0 | 2 | | | 0.212 | 0.21-0.39 | N | 21 | >68 | L | 0.21 | 0.1-0.38 | N |
| P07901# | 1985 | c.1397G>A | p.Arg466His | DGGE + Sanger | not | S | 6 | 11 | 1 | no | 4 | 0.277 | 0.21-0.39 | N | 25 | >68 | L | 0.03 | 0.1-0.38 | L |
| P07902 | 1997 | c.1397G>A | p.Arg466His | Sanger | not | S | 16 | 4 | 2 | yes | 5 | 0.388 | 0.21-0.39 | N | 61 | >68 | L | 0.06 | 0.1-0.38 | L |
| P07903 | 2007 | c.1397G>A | p.Arg466His | Sanger | not | A | | 1 | | | | 0.414 | 0.21-0.39 | H | 57 | >68 | L | 0.066 | 0.1-0.38 | L |
| P07904# | 1960 | c.1397G>A | p.Arg466His | DGGE + Sanger | not | S | 11 | 29 | 1 | yes | 4 | 0.369 | 0.21-0.39 | N | 64 | >68 | L | 0.07 | 0.1-0.38 | L |
| P07905# | 1982 | c.1397G>A | p.Arg466His | DGGE + Sanger | not | S | 11 | 14 | 62 | yes | 8 | 0.266 | 0.21-0.39 | N | 15 | >68 | L | 0.03 | 0.1-0.38 | L |
| P07906 | 2012 | c.1397G>A | p.Arg466His | Sanger | not | A | | 0 | | no | | 0.383 | 0.21-0.39 | N | 33 | >68 | L | 0.05 | 0.1-0.38 | L |
| P08001# | 1945 | c.120_121del | p.(Gly41Argfs*16) | DGGE + NGS | not | S | 22 | 55 | 1 | yes | 3 | 0.055 | 0.21-0.39 | L | 62 | >68 | L | 0.04 | 0.1-0.38 | L |
| P08002# | 1970 | c.120_121del | p.(Gly41Argfs*16) | DGGE + Sanger | not | S | 23 | 25 | 1 | yes | 3 | 0.026 | 0.21-0.39 | L | 21 | >68 | L | 0.02 | 0.1-0.38 | L |
| P08003# | 1968 | c.120_121del | p.(Gly41Argfs*16) | DGGE + NGS | not | S | 29 | 27 | | | | 0.075 | 0.21-0.39 | L | 49 | >68 | L | 0.06 | 0.1-0.38 | L |
| P08101# | 1952 | c.1397G>A | p.Arg466His | DGGE + Sanger | not | S | 26 | 46 | 2 | yes | 6 | 0.325 | 0.21-0.39 | N | 32 | >68 | L | 0.03 | 0.1-0.38 | L |
| P08201# | 1966 | c.160del | p.(Leu54Tyrfs*25) | DGGE + Sanger | not | S | 30 | 33 | 25 | yes | 5 | 0.036 | 0.21-0.39 | L | 30 | >68 | L | 0.02 | 0.1-0.38 | L |
| P08202 | 1986 | c.160del | p.(Leu54Tyrfs*25) | DGGE + Sanger | not | S | 1 | 13 | 86 | yes | 10 | 0.055 | 0.21-0.39 | L | 43 | >68 | L | 0.04 | 0.1-0.38 | L |
| P08203 | 2018 | c.160del | p.(Leu54Tyrfs*25) | Sanger | TRANS | S | 3 | 3 | 17 | | | 0.079 | 0.21-0.39 | L | 45 | >68 | L | 0.04 | 0.1-0.38 | L |
| P08301# | 1976 | c.548T>C | p.Leu183Pro | DGGE + Sanger | not | S | 18 | 21 | 13 | yes | 8 | 0.1 | 0.15-0.35 | L | 13 | >68 | L | 0.04 | 0.1-0.4 | L |
| P08401# | 1939 | c.1284_1285del | p.(Cys428Trpfs*44) | DGGE + Sanger | not | S | 20 | 58 | 30 | yes | 7 | 0.07 | 0.15-0.35 | L | 24 | >68 | L | 0.09 | 0.1-0.4 | L |
| P08402 | 1956 | c.1284_1285del | p.(Cys428Trpfs*44) | DGGE + Sanger | not | S | 20 | 51 | 24 | yes | | 0.05 | 0.15-0.36 | L | 34 | >68 | L | 0.06 | 0.14-0.35 | L |
| P08501# | 1992 | c.650del | p.(Gly217fs*15) | DGGE + Sanger | not | A | | 9 | | no | | 0.03 | 0.15-0.36 | L | 25 | >68 | L | 0.02 | 0.14-0.35 | L |
| P08601 | 1976 | EX1-6del | | MLPA | TRANS | S | 4 | 26 | 16 | yes | 9 | 0.06 | 0.15-0.36 | L | 38 | >68 | L | 0.04 | 0.14-0.35 | L |
| P08602 | 1956 | EX1-6del | | MLPA | not | S | 5 | 48 | 10 | yes | 5 | 0.06 | 0.15-0.36 | L | 45 | >68 | L | 0.05 | 0.14-0.35 | L |
| P08603 | 2000 | EX1-6del | | MLPA | not | S | 2 | 9 | 6 | yes | 6 | 0.04 | 0.15-0.36 | L | 29 | >68 | L | 0.03 | 0.14-0.35 | L |
| P08701 | 1986 | EX1-8del | | MLPA | not | S | 14 | 14 | 12 | no | 4 | 0.09 | 0.15-0.36 | L | 61 | >68 | L | 0.05 | 0.14-0.35 | L |
| P08801 | 1964 | EX5-6dup | | MLPA | not | S | 6 | 57 | | | | 0.03 | 0.15-0.36 | L | 55 | >68 | L | 0.03 | 0.14-0.35 | L |

**Table S1**: **Czech HAE Patients.** This table presents data on Czech patients diagnosed with Hereditary Angioedema (HAE) and provides the following information in the respective columns. **Sample ID**: Patients from the same family share the first three digits after 'P' in their Sample ID. Probands are distinguished by having a '1' in the last position of the Sample ID. [#] indicates previously published patients. **Causal variant**: The column lists the specific names of the causal variants identified at the cDNA level. **Protein change:** The corresponding alterations in the protein sequence resulting from the causal variants are described. **Method**: The column outlines the detection methods employed to identify the causal variants. The following techniques were used: DGGE - denaturing gradient gel electrophoresis was formerly used for variant screening; NGS - next generation sequencing covering almost the whole *SERPING1* including intronic parts was employed when other methods had not yielded results. MLPA - multiplex ligation-probe amplification was applied to detect large rearrangements; RA - restriction analysis was employed to confirm specific variants, typically in family members of a proband; Sanger - Sanger sequencing of exons and adjacent intronic parts has been the primary method of choice nowadays. **c.-21T>C** column provides information on the presence and form of the variant c.-21T>C in the patient. The following codes are used: not - the variant was not detected in the patient; TRANS - the variant is present in *trans* position to the causal variant; CIS - the variant is present in *cis* position to the causal variant; ND (not determined) - the c.-21T>C variant is present in the patient, but its specific form could not be determined; NA (not available) - DNA not available for an analysis. **Symptomatic/Asymptomatic** column indicates whether the patient is symptomatic (S), asymptomatic (A), or the information is not available (NA). **Age of onset** and **Age of diagnosis** columns contain the patient's age at the time of HAE onset or diagnosis, respectively. **No. of edema per year** column provides attack frequency. When multiple values were available for the patient, the maximum value was used for analysis. **Long term prophylaxis** presents the use of the treatment in the patient. **Clinical severity score**, as introduced by Bygum *et al.* [1], was calculated for each patient with available information on age of onset, attacks location, and long-term prophylaxis usage. Data from the Czech national registry covering the period 2012-2021 was used. **C1-INH [g/l]**, **C1-INH function [%]**, and **C4 [g/l]** indicate the measurements of C1-INH concentration, C1-INH function, and C4 concentration, respectively. These measurements were taken using various methods with different normal values, which are provided for each measurement in their respective columns. When multiple values were available for the patient, the lowest value was used for analysis. **Evaluation** of the measurements is given in the indicated columns using following codes: L – value lower than normal level; N – value within the normal range; H – value higher than normal level. † Patient P00401 carried the missense variant c.1361T>G, along with an in-frame duplication c.564_569dup, which we considered a variant of uncertain significance (VUS). Due to the limited compliance of the patient's family, it was not possible to determine whether the second variant is present in the *trans* or *cis* conformation. Patient P05301 and her daughter P05302 carried the pathogenic frameshift variant c.795_796delGGinsT, along with a VUS variant c.785T>C in cis conformation.

| Attack location | N |
|---|---|
| Abdominal | 2388 |
| Peripheral | 1542 |
| Abdominal + peripheral | 603 |
| Facial | 560 |
| Laryngeal | 314 |
| Other | 236 |
| Peripheral + other | 146 |
| Peripheral + laryngeal | 99 |
| Facial + peripheral | 83 |
| Abdominal + other | 75 |
| Facial + laryngeal | 49 |
| Abdominal + peripheral + other | 45 |
| Abdominal + facial | 40 |
| Abdominal + laryngeal | 29 |
| Abdominal + facial + peripheral | 24 |
| Laryngeal + other | 22 |
| Abdominal + peripheral + laryngeal | 19 |
| Facial + other | 13 |
| Peripheral + laryngeal + other | 6 |
| Abdominal + facial + laryngeal | 6 |
| Facial + peripheral + laryngeal | 6 |
| Facial + peripheral + other | 4 |
| Abdominal + facial + peripheral + other | 3 |
| Abdominal + facial + other | 1 |
| Abdominal + peripheral + laryngeal + other | 1 |
| Abdominal + laryngeal + other | 1 |
| Abdominal + facial + peripheral + laryngeal + other | 1 |
| Not known | 1 |

**Table S2**: **Location of HAE attacks** as they were recorded in 150 HAE patients in the Czech national registry of primary immunodeficiencies.

| Treatment | N | |
|---|---|---|
| Icatibant (Firazyr®) | 3350 | 62.93% |
| rhC1-INH (Ruconest®) | 799 | 15.01% |
| pnfC1-INH (Berinert®) | 902 | 16.95% |
| nfC1-INH (Cinryze®) | 79 | 1.48% |
| Attenuated androgens | 96 | 1.80% |
| Tranexamic acid | 88 | 1.65% |
| Frozen plasma | 8 | 0.15% |
| Kallikrein inhibitor | 1 | 0.02% |
| Total number of treated attacks | 5323 | |

**Table S3**: **Acute attack treatment.** Fixed drug doses were used in our cohort - Firazyr® (30mg), Ruconest® 2100 U 1-2 vials (median dose 2100 U, range 2100-4200 U), and Berinert® 500 IU 1-2 vials (median dose 1000 IU, range 500-1000 IU). Treatment had to be repeated in 800 attacks. The treatment was evaluated as effective in 98.6% of attacks.

## *Supplementary Methods*

**Databases and Bioinformatics**

Following population and variant databases and bioinformatic tools have been used to annotate variants and estimate variant impact.

*Population databases*

Genome Aggregation Database (GnomAD): https://gnomad.broadinstitute.org/ [2]

*Variant databases*

The Human Gene Mutation Database (HGMD) https://www.hgmd.cf.ac.uk/ [3]

ClinVar: www.ncbi.nlm.nih.gov/clinvar/ [4]

Leiden Open Variation Database (LOVD): https://www.lovd.nl/shared/variants/SERPING1 [5]

*Centralized databases for genetics and proteomics*

UCSC Genome Browser:https://genome.ucsc.edu/ [6]

Ensembl: http://www.ensembl.org [7]

Uniprot: https://www.uniprot.org/ [8]

*In Silico computational prediction tools*

Sorting Intolerant From Tolerant (SIFT): http://siftdna.org.submit.html [9]

PolyPhen (version 2): http://genetics.bwh.harvard.edu/pph2/ [10]

CADD: https://cadd.gs.washington.edu/ [11]

Exonic splicing enhancers: ESEfinder release 3.0 [12]

Human Splicing Finder, version 3.1: www.umd.be/hsf [13]

Maximum Entropy (MaxEnt) prediction model (MaxEntScan):

http://hollywood.mit.edu/burgelab/maxent/Xmaxentscan_scoreseq.html [14]

**RNA analysis**

Total RNA was extracted from the peripheral blood using RiboPure-Blood Kit (Thermo Fisher Scientific). Extracted RNA was reverse transcribed to cDNA with random hexamers using Superscript II (Thermo Fisher Scientific), following the manufacturer's instructions. Subsequent PCR was performed in two steps using Platinum SuperFi DNA polymerase (Thermo Fisher Scientific). Primer sequences are specified below, and reaction conditions are available upon request. Amplicons from the second reaction were checked on 2% agarose gels and characterized by capillary analysis through a commercial service (SeqMe). GeneScan™-1200 LIZ size standard was used for this purpose.

**Primers:**

1st step PCR:

| | |
|---|---|
| tag-C1rt-4a | CAGCACCTTTGTGGTTCTCATCGCCAGCCTCCTTACCCAGG |
| C1rt-4b | AGGGCTGAGAGCCTGTTCCA |

2nd step PCR:

| | |
|---|---|
| FAM-tag | FAM-CAGCACCTTTGTGGTTCTCA |
| C1rt-4b | AGGGCTGAGAGCCTGTTCCA |

**Minigene Assay**

To investigate the effect of sequence variant on RNA splicing, minigene constructs were created. Wild-type and mutant genomic fragments of SERPING1 comprising exon3 and 229 and 255 bp of flanking upstream and downstream introns were amplified using Platinum Pfx DNA polymerase (Thermo Fisher Scientific). The primer sequences are specified below, and reaction conditions are available upon request. PCR products were cloned into the multiple cloning sites inside the pET01 vector (MoBiTec) using restriction enzymes ApaI and SmaI.

HepG2 cells (European Collection of Authenticated Cell Cultures), maintained in RPMI 1640 medium (Sigma-Aldrich) supplemented with 10% foetal bovine serum (Sigma-Aldrich), were seeded (2 × 105 cells per transfection) into a 12-well plate 1 day prior to transfection. The cells were transfected using 500 ng of minigene construct and 2.4µl of transfection reagent XtremeGene 9 (Roche). RNA was extracted 24 h after transfection using a Quick-RNA Miniprep Kit (Zymo Research), and subsequently, RT-PCR was performed. Reverse transcription was performed using SuperscriptTM II reverse transcriptase (Thermofisher Scientific) according to the manufacturers' protocol. Subsequent PCR for capillary electrophoresis was performed using Taq DNA polymerase (Thermofisher Scientific) and fluorescently labelled primer pET1A-FAM.

Primers:

PCR - capillary analysis

| | |
|---|---|
| pET1A-FAM | CAGCACCTTTGTGGTTCTCA |
| rPET_PS02 | GCACTGATCCACGATG |

SERPING1 cloning

| | |
|---|---|
| f_C1e3_clon_ApaI | GGGCCCTTCTGCAGAGCACATTCCTGT |
| r_C1e3_clon_SmaI | CCCGGGGGTTAGTGGCTGCGACCTTA |

SERPING1 mutagenesis

| | |
|---|---|
| f_C1e3_c.550+3A>G_mut | ccaggtcctgctcggtgagaccctgc CCAGGTCCTGCTCGGTGAGACCCTGC |
| r_C1e3_c.550+3A>G_mut | GCAGGGTCTCACCGAGCAGGACCTGG |

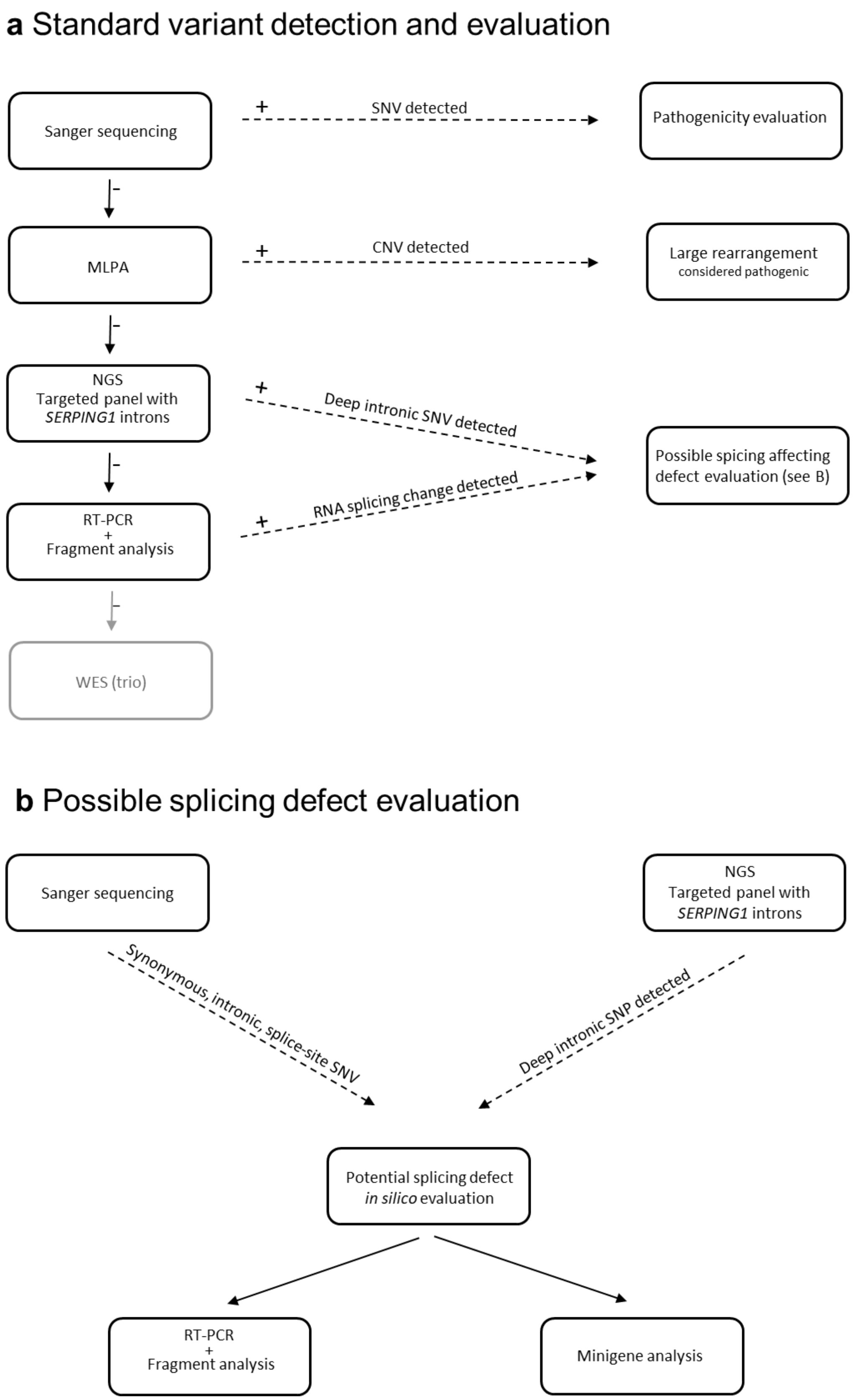

a Standard variant detection and evaluation
Sanger sequencing
+
SNV detected
Pathogenicity evaluation
-
MLPA
+
CNV detected
Large rearrangement
considered pathogenic
-
NGS
Targeted panel with
*SERPING1* introns
+
Deep intronic SNV detected
Possible spicing affecting
defect evaluation (see B)
-
RT-PCR
+
Fragment analysis
+
RNA splicing change detected
-
WES (trio)
b Possible splicing defect evaluation
Sanger sequencing
NGS
Targeted panel with
*SERPING1* introns
Synonymous, intronic, splice-site SNV
Deep intronic SNP detected
Potential splicing defect
*in silico* evaluation
RT-PCR
+
Fragment analysis
Minigene analysis

**Fig. S1: The current workflow for screening pathogenic variants in SERPING1.**'+' indicates the presence of a detected variant, while '-' indicates its absence. Solid line arrows represent the procedural steps, while hatch-marked arrows indicate the need for pathogenicity evaluation of the detected variant. **a** Standard variant detection and evaluation. Initially, Sanger sequencing is performed on exonic and adjacent intronic regions. If no potentially pathogenic variant is identified, MLPA is conducted. Two methods are employed to screen for splicing and deep intronic defects: Targeted NGS and RT-PCR with fragment analysis. Targeted NGS covers almost all intronic regions of SERPING1 and enables the detection of copy number variations (CNVs). Although the MLPA step could theoretically be omitted, it is typically performed due to its speed and efficiency in CNV detection. Concurrently, RNA analysis by RT-PCR is sometimes performed if patient RNA can be obtained promptly. Finally, in one case where no pathogenic variant was detected by previous methods, WES analysis is currently performed on the patient and their parents. While the pathogenicity of null variants is often clear, further evaluation is required for missense and splicing variants. **b** Possible splicing defect evaluation. If RT-PCR analysis has not been performed during the standard evaluation, a set of RT-PCRs with primers located inside exons, followed by fragment analysis, is conducted. In parallel with the RT-PCR analysis, or as an alternative in cases where patient RNA is not available, minigene analysis is utilized.

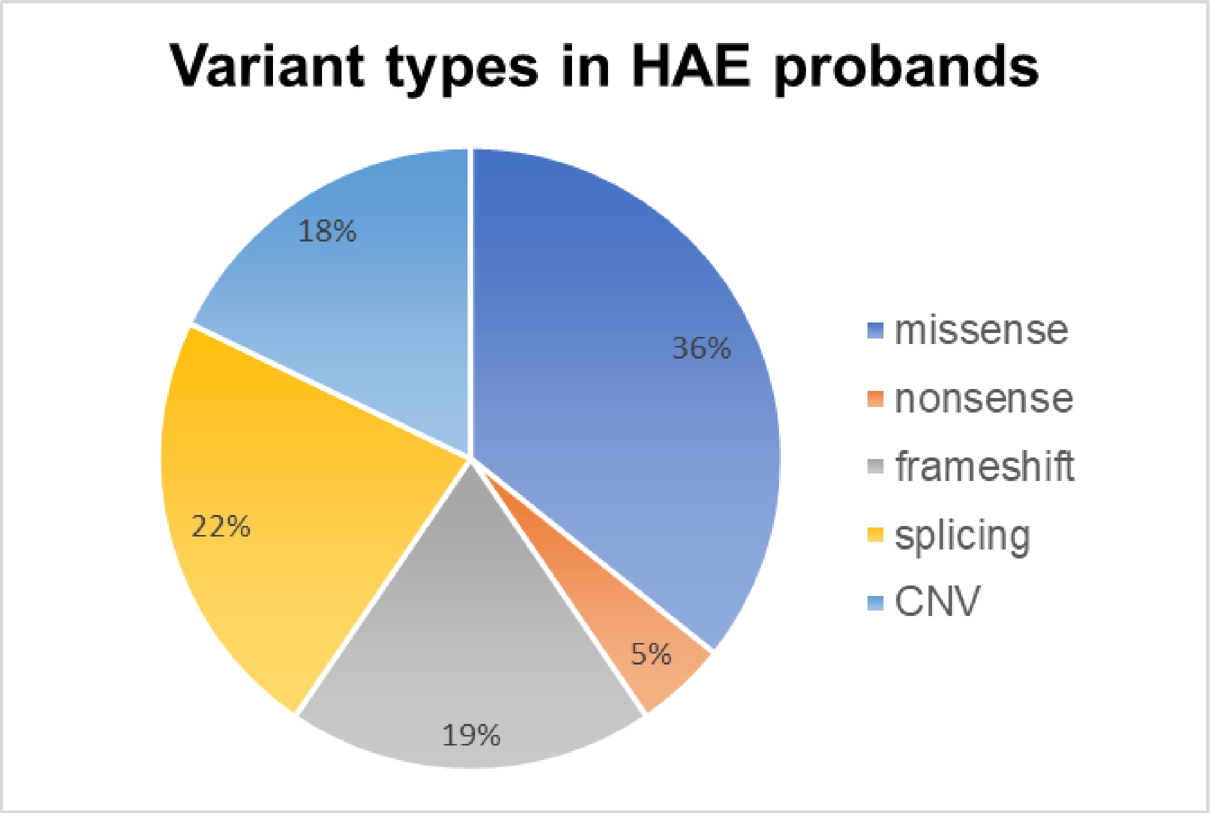


**Fig. S2: Proportions of probands with indicated causal variant type in our cohort.** The proportions of probands with detected causal variant types were calculated from 84 out of 85 pedigrees in our cohort. In one case, no causal variant was identified. The overall SERPING1 variants comprise missense variants (36%; Table 2) listed in Table 2, nonsense variants (5%; Table 3) and frameshift variants (19%; Table 4), splicing defects (22%; Table 5) and large deletions and duplications (18%; Table 6) amounting to a total of 56 distinct variants and including 5 novel variants.

*References*

1. Bygum A, Fagerberg CR, Ponard D, Monnier N, Lunardi J, Drouet C. Mutational spectrum and phenotypes in Danish families with hereditary angioedema because of C1 inhibitor deficiency. Allergy [Internet]. 2011;66:76–84. Available from: https://doi.org/10.1111/j.1398-9995.2010.02456.x

2. Karczewski KJ, Francioli LC, Tiao G, Cummings BB, Alföldi J, Wang Q, et al. The mutational constraint spectrum quantified from variation in 141,456 humans. Nature [Internet]. 2020 [cited 2022 Nov 30];581:434–43. Available from: https://doi.org/10.1038/s41586-020-2308-7

3. Stenson PD, Mort M, Ball EV, Evans K, Hayden M, Heywood S, et al. The Human Gene Mutation Database: towards a comprehensive repository of inherited mutation data for medical research, genetic diagnosis and next-generation sequencing studies. Hum Genet [Internet]. 2017 [cited 2022 Mar 8];136:665–77. Available from: https://www.ncbi.nlm.nih.gov/pmc/articles/PMC5429360/

4. Landrum MJ, Lee JM, Benson M, Brown GR, Chao C, Chitipiralla S, et al. ClinVar: improving access to variant interpretations and supporting evidence. Nucleic Acids Res [Internet]. 2018 [cited 2022 Nov 30];46:D1062–7. Available from: https://doi.org/10.1093/nar/gkx1153

5. Fokkema IFAC, Taschner PEM, Schaafsma GCP, Celli J, Laros JFJ, den Dunnen JT. LOVD v.2.0: the next generation in gene variant databases. Human Mutation [Internet]. 2011 [cited 2022 Nov 30];32:557–63. Available from: https://doi.org/10.1002/humu.21438

6. Kent WJ, Sugnet CW, Furey TS, Roskin KM, Pringle TH, Zahler AM, et al. The Human Genome Browser at UCSC. Genome Res [Internet]. 2002 [cited 2022 Nov 30];12:996–1006. Available from: https://doi.org/10.1101/gr.229102

7. Cunningham F, Allen JE, Allen J, Alvarez-Jarreta J, Amode MR, Armean IM, et al. Ensembl 2022. Nucleic Acids Research [Internet]. 2022 [cited 2022 Nov 30];50:D988–95. Available from: https://doi.org/10.1093/nar/gkab1049

8. The UniProt Consortium. UniProt: the universal protein knowledgebase in 2021. Nucleic Acids Research [Internet]. 2021 [cited 2022 Nov 30];49:D480–9. Available from: https://doi.org/10.1093/nar/gkaa1100

9. Vaser R, Adusumalli S, Leng SN, Sikic M, Ng PC. SIFT missense predictions for genomes. Nat Protoc [Internet]. 2016 [cited 2022 Nov 30];11:1–9. Available from: https://doi.org/10.1038/nprot.2015.123

10. Adzhubei I, Jordan DM, Sunyaev SR. Predicting Functional Effect of Human Missense Mutations Using PolyPhen-2. Curr Protoc Hum Genet [Internet]. 2013 [cited 2022 Nov 30];0 7:Unit7.20. Available from: https://doi.org/10.1002/0471142905.hg0720s76

11. Rentzsch P, Witten D, Cooper GM, Shendure J, Kircher M. CADD: predicting the deleteriousness of variants throughout the human genome. Nucleic Acids Research [Internet]. 2019;47:D886–94. Available from: https://doi.org/10.1093/nar/gky1016

12. Cartegni L, Wang J, Zhu Z, Zhang MQ, Krainer AR. ESEfinder: a web resource to identify exonic splicing enhancers. Nucleic Acids Res [Internet]. 2003 [cited 2022 Nov 30];31:3568–71. Available from: https://doi.org/10.1093/nar/gkg616

13. Desmet FO, Hamroun D, Lalande M, Collod-Bëroud G, Claustres M, Béroud C. Human Splicing Finder: an online bioinformatics tool to predict splicing signals. Nucleic Acids Research [Internet]. 2009;37:e67. Available from: https://doi.org/10.1093/NAR/GKP215

14. Yeo G, Burge CB. Maximum Entropy Modeling of Short Sequence Motifs with Applications to RNA Splicing Signals. https://home.liebertpub.com/cmb [Internet]. 2004;11:377–94. Available from: https://doi.org/10.1089/1066527041410418
